# Active mid-infrared ring resonators

**DOI:** 10.1038/s41467-023-44628-7

**Published:** 2024-01-19

**Authors:** Dmitry Kazakov, Theodore P. Letsou, Maximilian Beiser, Yiyang Zhi, Nikola Opačak, Marco Piccardo, Benedikt Schwarz, Federico Capasso

**Affiliations:** 1 Harvard John A. Paulson School of Engineering and Applied Sciences, Harvard University, Cambridge, MA 02138 USA; 2 Department of Electrical Engineering and Computer Science, Massachusetts Institute of Technology, Cambridge, MA 02142 USA; 3 Institute of Solid State Electronics, TU Wien, 1040 Vienna, Austria; 4 UC Berkeley, Berkeley, CA 94720 USA; 5 Department of Physics, Instituto Superior Técnico, Universidade de Lisboa, 1049-001 Lisbon, Portugal; 6 Instituto de Engenharia de Sistemas e Computadores - Microsistemas e Nanotecnologias (INESC MN), 1000-029 Lisbon, Portugal

**Keywords:** Mid-infrared photonics, Microresonators, Quantum cascade lasers, Integrated optics

## Abstract

High-quality optical ring resonators can confine light in a small volume and store it for millions of roundtrips. They have enabled the dramatic size reduction from laboratory scale to chip level of optical filters, modulators, frequency converters, and frequency comb generators in the visible and the near-infrared. The mid-infrared spectral region (3−12 μm), as important as it is for molecular gas sensing and spectroscopy, lags behind in development of integrated photonic components. Here we demonstrate the integration of mid-infrared ring resonators and directional couplers, incorporating a quantum cascade active region in the waveguide core. It enables electrical control of the resonant frequency, its quality factor, the coupling regime and the coupling coefficient. We show that one device, depending on its operating point, can act as a tunable filter, a nonlinear frequency converter, or a frequency comb generator. These concepts extend to the integration of multiple active resonators and waveguides in arbitrary configurations, thus allowing the implementation of purpose-specific mid-infrared active photonic integrated circuits for spectroscopy, communication, and microwave generation.

## Abstract (web summary)

Multifunctional active mid-infrared ring resonators and directional couplers with quantum cascade laser cores allow electrical control of resonant frequency and quality factors, tunable filtering and frequency comb generation.

## Introduction

On-chip integration of photonic components has become key to scaling down optical laboratory experiments and transferring them to portable commercial technologies^1–5^. Photonic integrated circuits (PICs) — optical counterparts of CMOS integrated circuits — may enable a reduction of global electricity consumption by data centers interconnects^6^, fast and efficient classical and quantum optical signal processors^7–9^, and provide lightweight, cost-effective devices for spectroscopy above the ground and in open space^10,11^.

The most versatile building blocks of PICs are ring resonators. They form the basis of optical notch filters^12,13^, modulators^14^, frequency converters^15^, and frequency comb generators^16,17^. Active and passive ring resonators in the near-infrared (near-IR) and in the visible range have been long and widely available either as standalone elements or as PIC components^18–21^. The mid-IR range, despite being of utmost interest for spectroscopy, chemical and biological sensing, and free-space communications, does not yet fully benefit from integrated photonic solutions. There, the historically long absence of compact and powerful laser sources substantiated lack of motivation to develop passive waveguides, resonators, and, ultimately, integrated photonic chips. Simultaneously, the materials with well-established processing techniques such as SiO_2_ and Si_3_N_4_ employed for the near-IR and the visible range^22^, demonstrate prohibitively large losses towards longer wavelengths, which requires usage of unconventional materials, such as germanium, chalcogenides, or halide crystals with less mature fabrication protocols. The development of interband cascade laser (ICL) and quantum cascade laser (QCL) epitaxial growth and fabrication technologies has provided powerful monolithic laser sources across the 3–12 μm range, that operate under direct current (DC) driving at room temperature. The availability of these light sources sparked efforts towards photonic integration in the mid-IR with numerous demonstrations of integrated waveguides and high quality factor passive ring resonators^23–27^.

Here we demonstrate active ring resonators based on QCL gain regions that operate in the mid-IR, specifically around 8.2 μm — in the atmospheric transparency window. We show that the same device, depending on the way it is driven, can act as a filter, wavelength converter, or a frequency comb generator. Active ring QCL resonators thus have the potential of serving as reconfigurable building blocks of larger-scale agile hybrid mid-IR PICs. This demonstration is not wavelength-specific and can be readily extended to the entire mid-IR range, utilizing the complete spectral gamut of ICLs and QCLs.

## Results

**Fig. 1:**
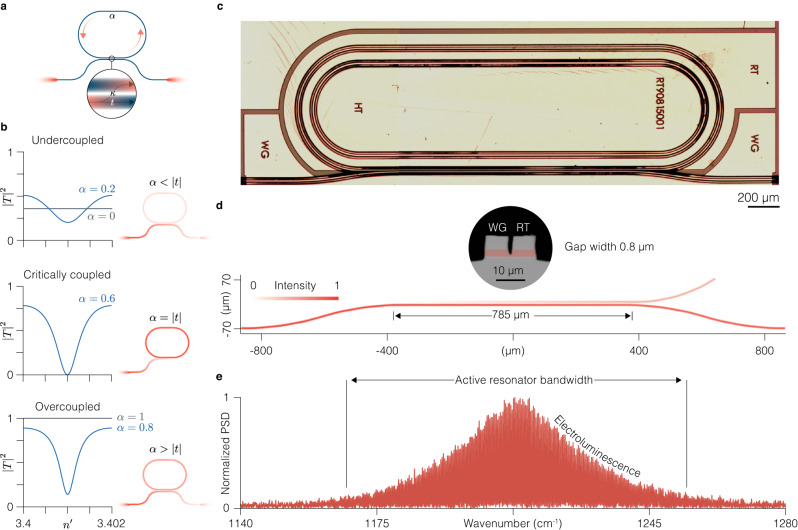
Ring quantum cascade resonators with active directional couplers. **a** Schematic of a ring resonator with a directional coupler. Light injected through the left waveguide facet gets partially coupled into the ring resonator and partially transmitted on to the right output facet. Upon propagation within the resonator, the field experiences gain or attenuation depending on the value of *α*. **b** Theoretical intensity transmission of the ring resonator with a tunable refractive index ${n}^{{\prime} }$ and the roundtrip loss coefficient *α* in the three coupling regimes, after Eq. (1). The depictions to the right of the transmission curves schematize the light intensity distribution in the waveguide and the ring when the refractive index is such that the resonance condition is fulfilled (${n}^{{\prime} }L/\lambda$ is an integer). In this example we assumed *λ* = 8 *μ*m and resonator length *L* = 4 mm. ${\left|t\right|}^{2}=1-{\left|\kappa \right|}^{2}$ is the power transmission coefficient. **c** Optical microscope image of the racetrack quantum cascade (QC) resonator with an integrated active directional coupler. Contact sections for separate biasing of the integrated components are denoted with RT, for the racetrack, WG, for the waveguide coupler and HT, for the integrated heater. In this work the heater remained unbiased. **d** Simulated light intensity distribution (*λ* = 7.9 *μ*m, *n*_RT_ = 3.323) in the coupling region when the light is launched from the left port of the directional coupler. The inset shows an optical microscope image of the cross-section of the coupling region. For clarity, the waveguide cores filled with the QC gain medium are false-colored in red. **e** Experimental spectrum of the subthreshold emission (electroluminescence) of a Fabry-Perot QC laser fabricated from the same epitaxial material as the ring resonator shown in **c**. The spectral band occupied by the electroluminescence signal corresponds to the region where *α* can be tuned, hence defining the optical bandwidth of the active QC resonators. PSD, power spectral density.

We start by reviewing the operating principle of an active ring resonator. The injection of light into the ring resonator can be achieved with several different coupler geometries. Here we restrict our focus to directional couplers where coupling occurs via an evanescent wave from a waveguide separated by a narrow gap from the resonator sidewall (Fig. 1a). The width of the gap, the index of the material in the gap, the length of the coupling region, the resonator length and its intrinsic loss define the amount of light coupled into the resonator at a given wavelength, as well as its amplitude and phase response on resonance.

The ability to tune these parameters allows the implementation of integrated ring resonators with control over the resonance frequency and the coupling strength between the waveguide and the resonator, quantified by the power coupling coefficient ∣*κ*∣^2^ (with the amplitude coupling coefficient given by $\left|\kappa \right|$). In passive resonators, comprised of low-loss waveguides based on transparent dielectrics, the resonance frequency can be adjusted, or modulated at kilohertz rates by the thermal tuning of a purely real refractive index *n*, and at gigahertz rates using the electro-optic effect^28–31^. Active ring resonators are even more versatile, as they incorporate not a transparent, but an amplifying medium in the waveguide core. The optical index $n={n}^{{\prime} }+i{n}^{{\prime\prime} }$ is thus complex, and both the real refractive index ${n}^{{\prime} }$ and the extinction coefficient $n^{{\prime\prime} }$ are tunable parameters. Absorption ($n^{{\prime\prime} }$ > 0) can be turned into gain ($n^{{\prime\prime} }$ < 0) by achieving population inversion via electrical or optical pumping. Therefore, not only the resonance frequency and the coupling strength, but also the intrinsic quality factor of the resonator can be tuned. To visualize the effects of the complex index *n* on the characteristics of such active ring resonator it is instructive to examine its intensity transmission ${\left|T\right|}^{2}$ ^32,33^:

1 $${\left|T\right|}^{2}=\frac{{\alpha }^{2}+{\left|t\right|}^{2}-2\alpha \left|t\right|\cos \left(2\pi {n}^{{\prime} }L/\lambda \right)}{1+{\alpha }^{2}{\left|t\right|}^{2}-2\alpha \left|t\right|\cos \left(2\pi {n}^{{\prime} }L/\lambda \right)}$$

In Eq. (1) *L* is the resonator circumference, ${\left|t\right|}^{2}=1-{\left|\kappa \right|}^{2}$ quantifies the portion of the field intensity that is not coupled to the ring resonator and transmitted directly through the waveguide coupler, *λ* is the vacuum wavelength of the input optical field, and $\alpha=\exp (-2\pi {n}^{{\prime\prime} }L/\lambda )$ is the residual fraction of the field amplitude after one resonator roundtrip^32^. When 0 ≤ *α* < 1 there is a net roundtrip loss. When *α* = 1 the resonator is lossless. In the case *α* > 1 there is net roundtrip gain. Here *α* aggregates the fixed contributions from the internal dissipation mechanisms, such as sidewall roughness scattering, bending loss, material absorption, and the variable contribution from the active medium in the form of gain or absorption, depending on population inversion.

Figure 1b illustrates, in accordance with Eq. (1), how the intensity transmission ${\left|T\right|}^{2}$ of an active resonator can be modified by tuning *n*. The detuning of the input field from the cavity resonance — that enters Eq. (1) through the cosine term — is controlled via the real part of the refractive index (equivalently, the detuning from the resonance can be controlled by changing the wavenumber *k* = 2*π*/*λ*). On resonance, when ${n}^{{\prime} }L/\lambda$ is an integer (in this example *λ* = 8 *μ*m, *L* = 4 mm), the transmission shows a characteristic dip — the result of partial destructive interference between the portion of the field transmitted through the waveguide coupler and the portion of the field coupled to the ring and back to the waveguide. When $\alpha < \left|t\right|$, the net internal resonator loss, quantified by 1 − *α*^2^, is larger than the external coupling loss, equal to ${\left|\kappa \right|}^{2}$, and the system is said to be undercoupled. An extreme case of undercoupling — effectively, no coupling at all — occurs when the attenuation is total (*α* = 0): then the transmission is simply given by ${\left|T\right|}^{2}={\left|t\right|}^{2}$ and does not show any resonant behavior, as if there were no ring resonator next to the waveguide, but just a loss channel defined by the power coupling coefficient ${\left|\kappa \right|}^{2}$. In the critical coupling regime at $\alpha=\left|t\right|$ the destructive interference is perfect as the resonator mode loses as much power to internal dissipation as due to coupling back to the waveguide. Overcoupling occurs when $\alpha \, > \, \left|t\right|$, such that the net internal resonator loss is smaller than the external coupling loss. When *α* = 1, the resonator is transparent, which is an extreme case of overcoupling that can only occur in active resonators.

Each of the coupling regimes is useful on its own. Due to a large extinction ratio on the resonance, the critical coupling is advantageous for sharp notch filters and intensity modulators. Overcoupled ring resonators, due to a sharp *π* phase change across the resonance and a reduced resonance contrast, can be used as phase modulators^14^. In wavelength converters based on optical parametric oscillators overcoupling maximizes the conversion efficiency from the pump to the signal and the idler waves^34^. While in passive resonators, each of these functions is achieved by fabricating them to perform in a certain coupling regime with a fixed value of ${\left|\kappa \right|}^{2}$ and *α*, in active resonators, the regime can be set on demand by tuning *α*. In what follows we show active ring resonators with a tunable complex index *n* that enables control over the resonance frequency and the coupling regime.

### Active resonator design and working principle

Operation in the mid-IR is enabled by InGaAs waveguides with a low doped InP cladding with the core formed by a QCL active region^35^. Parameter tuning can thus be achieved via electrically variable optical gain associated with intersubband transitions. We used an InP wafer with an epitaxially grown active material on which we fabricated ring resonators shaped as racetracks (RT) with integrated evanescent wave directional couplers using a standard ridge process (Methods). The coupler waveguide (WG) is separated by a narrow air gap from the straight section of the racetrack (Fig. 1c). The power coupling coefficient ${\left|\kappa \right|}^{2}$ is set at the design stage by the length of the interaction region and by the width of the air gap (Fig. 1d). For a gap width of 0.8 μm, attainable using optical lithography, we simulate a power coupling ratio of −10 dB (Supplementary Fig. S1). The fabricated devices feature slightly underetched gaps, which translates into a coupling coefficient higher than its design value (Fig. 1d). Both the WG and the RT contain the QC gain medium and have separate contacts that enable independent electrical driving. The WG coupler is thus active as well, which offers two benefits. First, separate control over the injected electrical currents into the WG and the RT allows for fine tuning of the coupling strength, as asymmetric electrical driving results in a mismatch of mode indices inside the WG and RT (Supplementary Sections I and III). Second, the WG driven above its transparency current can act as an independent optical amplifier for the input and output wave, thus compensating for insertion loss or even providing net amplification. Devices reported here operate around the design wavelength of 8.2 *μ*m and have an optical bandwidth of about 50 cm^−1^ as seen from the spectrum of the amplified spontaneous emission of a Fabry-Perot (FP) QCL fabricated on the same wafer and driven below its lasing threshold (Fig. 1e).

### Regime control and tunability of active QC resonators

**Fig. 2:**
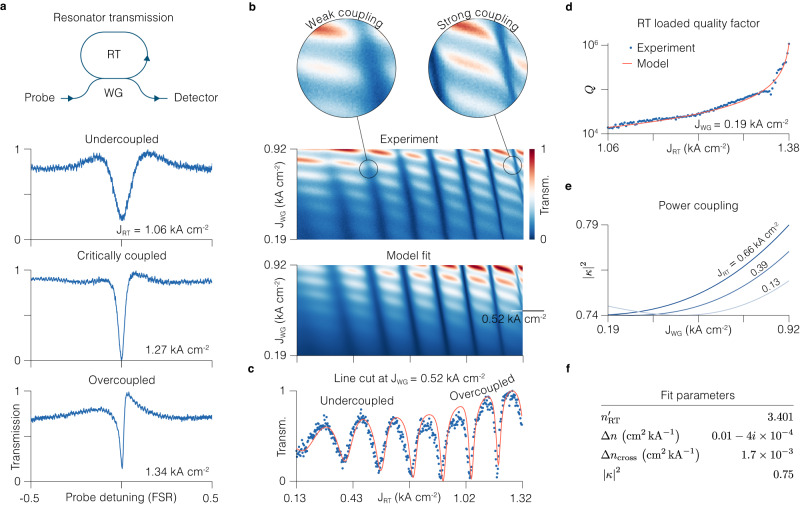
Transmission characteristics of the racetrack-waveguide system. **a** Experimental transmission of the RT coupled to the WG as the wavelength of the probe laser is swept across the RT resonance. The resonance narrows for increasing RT current density. The asymmetric resonance in the overcoupled regime has a pronounced Fano lineshape (Methods). **b** Transmitted intensity of the probe signal at 1222 cm^−1^ as function of current density of the racetrack (RT) and the directional waveguide coupler (WG) and the corresponding least squares model fit. The experiment shows the ability to control complex mode indices in the coupled resonators. The independent control over quality factors of both WG and FP allows traversing different regimes of resonances, such as weak and strong coupling^37^. Strong coupling regime manifests itself in the avoided crossing in the resonances. **c** Experimental transmission (dots) and model fit (solid line) as function of the RT current density for the fixed WG current density of 0.52 kA cm^−2^. See Supplementary Section III for the details on the model and the fitting. **d** Extracted (dots) and modeled (solid line) RT loaded quality factor (*Q*) as function of the RT current. We extract *Q* by fitting the individual resonances, as shown in **a**, with a Fano model, for each RT current setting (see Methods). **e** Power coupling coefficient, calculated with Eq. (2) based off the extracted experimental values for the complex indices of the WG and RT, as function of the WG current for three different values of the RT current. **f** Values extracted from the fit shown in **b** and **c**. *n*_RT_, background mode index (when the electrical pumping is off) of the RT. Δ*n*, complex index change per unit of current density. Δ*n*_cross_, index change due to thermal crosstalk between WG and RT. ∣*κ*∣^2^, power coupling coefficient.

To demonstrate the ability to tune the parameters of the active RT resonator, and to set its operation regime, we measure the transmission of the RT-WG system while keeping the RT below its lasing threshold. First we sweep the wavelength of a probe laser across one RT resonance while keeping the WG bias constant. The transmission as a function of probe detuning shows a dip, whose linewidth decreases as we increase the electrical pumping of the RT — the resonator quality factor grows as the population inversion increases (Fig. 2a). In this way the RT quality factor *Q* can be continuously tuned over two orders of magnitude (Fig. 2d). According to Eq. (1), in this way we can set the coupling regime at will.

Next, we fix the probe laser at 1222 cm^−1^ and sweep the drive currents of both the WG and the RT (Fig. 2b). Increasing the RT current leads to several effects in the recorded transmission. First, the thermal (due to resistive heating) and carrier-induced change of the real part of the refractive index effectively sweeps the RT cavity mode resonances with respect to the fixed probe laser frequency (Fig. 2b, c). Second, as stated previously, increasing pumping increases population inversion in the RT active medium, which decreases the loss (increasing the imaginary part of the refractive index) and leads to the narrowing of the resonances (Fig. 2b, c). Third, the mode index mismatch between the coupled waveguides caused by the asymmetric driving of the WG and the RT leads to the change of the power coupling coefficient ${\left|\kappa \right|}^{2}$ between the WG and the RT (Fig. 2e) according to (Supplementary Section I)

2 $${\left|\kappa \right|}^{2}=\frac{1}{1+{(\delta /k)}^{2}}{\sin }^{2}\left(\frac{\pi \sqrt{{k}^{2}+{\delta }^{2}}}{\lambda }{L}_{{{{{{{{\rm{int}}}}}}}}}\right)$$

where $\delta={n}_{{{{{{{{\rm{RT}}}}}}}}}^{{\prime} }-{n}_{{{{{{{{\rm{WG}}}}}}}}}^{{\prime} }$ is the real index mismatch in the coupled waveguides, *k* is the coupling strength defined by the gap and index of the material it is filled with, and *L*_int_ is the length of the interaction region.

Not captured by Eq. (1) is the finite reflectivity (*R* ≈ 0.3) of the cleaved WG facets, which has an impact on the transmission. When the WG is biased at or below transparency the system is comprised by a low-finesse FP resonator (WG) coupled to a high-finesse RT resonator. The resonant structure of the transmission thus appears as well when sweeping the current of the WG (Fig. 2b). At low WG bias, its resonant structure is washed out due to high absorption in absence of population inversion. Increasing the WG bias leads to a decrease in absorption. At high bias of the WG and RT, both resonators attain a high finesse and the resonance profiles show avoided mode crossing — a signature of strongly coupled resonators (Fig. 2b)^36^. When the WG has a low bias (and hence a low quality factor), the coupling to the high-quality-factor RT leads to an asymmetric Fano resonance lineshape, visible in the overcoupled regime in Fig. 2a (see Methods for further details on the Fano resonance lineshape)^37^.

The transmission characteristics of the RT-WG system agrees remarkably well with a simple transfer matrix model that we adapt from ref. ^38^ to account for the complex mode indices and for the thermal crosstalk between the coupled waveguides (Fig. 2b, c and Supplementary Section III). The model captures all features of the experimental transmission. By fitting the model to the experimental data we can extract relevant device parameters, such as the effective refractive index (*n*_RT_) in absence of biasing, its change per unit of current density, the power coupling coefficient ${\left|\kappa \right|}^{2}$ between the RT and the WG and the strength of thermal crosstalk (Fig. 2f).

### Tunable nonlinear frequency converter

**Fig. 3:**
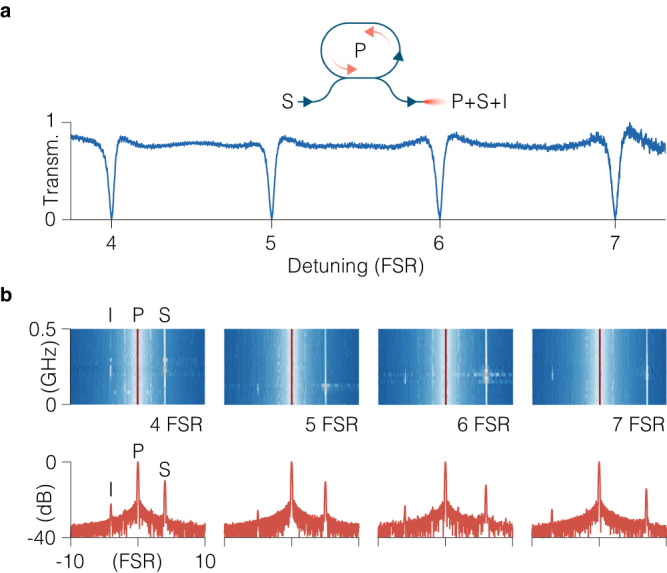
Four-wave mixing in ring QCLs above the threshold. **a** Experimental transmission, below the lasing threshold, of the ring resonator, showing four consecutive resonances on the blue side of the pump wave (P) generated by the ring QCL, into which we inject signal light (S) to generate idler sidebands (I) by four-wave mixing. **b** Top graphs are the experimental spectrograms of the ring QCL above the threshold under external optical injection as the detuning of the signal is swept across the ring resonance, shown for four subsequent resonances, 4, 5, 6 and 7 FSRs to the blue of the ring lasing frequency. Bottom graphs depict optical spectra for the detuning of the signal wave when the idler wave is the strongest.

Next we show the utility of the ring resonator as a nonlinear optical frequency conversion element. We bring the RT above the lasing threshold, pumping it strong enough that the internal gain compensates the loss through the coupler (such setting corresponds to a condition in Eq. (1) when $\alpha \, > \, 1/\left|t\right|$). In the lasing regime the ring resonator generates a strong single-frequency unidirectional intracavity field (note that the lasing frequency of the RT is different from the lasing frequency of the probe laser; the RT lases on the resonance at the center of its gain peak, whereas probing is done on a resonance away from the gain peak). The RT field serves as a pump for the parametric gain experienced by the externally injected weak probe signal whose frequency is detuned from the frequency of the intracavity field (Fig. 3a)^39^. We sweep the wavelength of the injected signal through four adjacent resonances of the RT resonator, on the blue side of the RT pump field (Fig. 3a). When tuning into each of the four resonances we observe an appearance of the idler sideband symmetrically on the red side of the pump — a signature of parametric amplification via four-wave mixing (FWM) provided by the coherent interaction of the pump and the signal waves (Fig. 3b)^40^. Here FWM is degenerate, with two pump photons being converted to a signal and an idler photon with the frequencies of the four interacting waves related by 2*ω*_P_ = *ω*_S_ + *ω*_I_.

### Ring frequency combs with active couplers

**Fig. 4:**
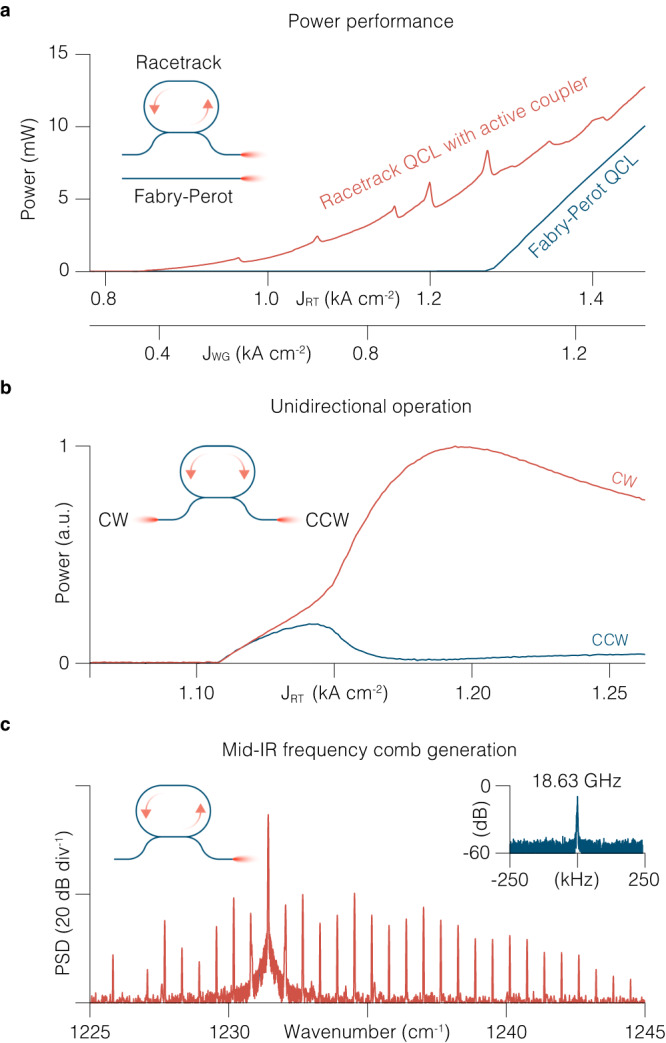
Ring QCL frequency comb with active couplers. **a** Output power from the front waveguide facet of the ring QCL above the threshold as both RT and WG currents are swept simultaneously. Both WG and RT are operated under continuous wave electrical current injection (Methods). The peaks in the RT emission arise due to the Fabry-Perot resonances of the WG, as its current is ramped up. Shown for comparison, output power of a Fabry-Perot (FP) QCL fabricated on the same wafer as the ring QCL. While FP QCL emits from both facets, the graph shows the power collected from one facet only. **b** Experimental intensities as function of the injected current collected simultaneously from both WG ports on two external detectors, showing the regimes of bidirectional and unidirectional lasing. **c** Experimental spectrum of the self-starting frequency comb in a ring QCL, when it operates in a unidirectional regime. The spectrum is acquired with a custom-built Fourier Transform Infrared Spectrometer. PSD, power spectral density. Inset shows a corresponding RF spectrum of the intermode beat note. RBW is 750 Hz, sweep time is 1 s.

Finally we remove the external probe laser and characterize ring QCLs with active couplers as standalone lasers and self-starting frequency comb generators. FP QCLs have established themselves as compact mid-IR and THz frequency comb generators^41–45^. Recently, ring QCLs gained momentum as alternative sources of mid-IR frequency combs, with the promise of better stability, higher power efficiency and larger spectral coverage than their FP counterparts^46–50^. The main disadvantage of ring QCLs with respect to FP QCLs so far has been the low outcoupling efficiency of the generated radiation which limited the output power to submilliwatt levels, making them largely inferior to FP QCLs that feature power levels in the 10–100 mW range^46,47^. In this regard, RT QCLs with directional couplers are superior to ring QCLs without coupling ports, in that they enable the extraction of optical power at levels that are suitable for applications. The RT QCLs presented here feature output power levels above 10 mW at room temperature, on par with FP QCLs of similar length and identical waveguide width fabricated on the same wafer (Fig. 4a). The high output power is enabled by the large coupling coefficient, as well as by the active directional coupler that can further amplify the outcoupled radiation when biased above the transparency.

A distinguishing feature of ring QCLs is that the single-mode instability mechanism that triggers the frequency comb formation is different from FP QCLs and leads to a number of laser states that are not accessible in FP QCLs, such as homoclons, Turing rolls, and solitons^46,49,51^. The prerequisite for the formation of such states is for the lasing field to propagate unidirectionally inside the resonator, which cannot be fulfilled in a FP cavity, but is possible in a ring resonator^46^. While circularly symmetric ring QCLs present an ideal condition for unidirectional operation in absence of strong backscattering, RT QCLs can feature higher backscattering coefficients due to reflections off the uncoated facets of the WG coupler and the discontinuity of the effective refractive index at the transition from the straight to the circular RT section, potentially leading to complex dynamics of the counter-propagating waves^52^. Nevertheless RT QCLs shown here operate in a unidirectional regime above the threshold, as we show by simultaneously detecting the light intensity coming out of both ends of the WG coupler while increasing the RT pump current (Fig. 4b). Right above the threshold, clockwise (CW) and counterclockwise (CCW) waves grow at same rate, up until the point where CW wave intensity starts growing at a higher rate at the expense of the decreasing intensity of the CCW wave, ultimately leading to lasing in CW direction only at higher pumping level (Fig. 4b). As in circular ring QCLs once the parametric gain provided by the single-mode intracavity pump field is high enough, phase turbulence leads to a single-mode instability resulting in the formation of a frequency comb with a characteristic bell-shaped spectral envelope (Fig. 4c)^51^. An in-depth theoretical and experimental investigation of frequency comb states and the underlying nonlinear dynamics of their formation is discussed in ref. ^53^, a study made possible by the active waveguide technology presented in this paper.

## Discussion

The demonstrated active ring resonators, thanks to the ability to control coupling, resonance detuning, and quality factors below the lasing threshold, as well as their inherently giant *χ*^(3)^ optical nonlinearity, of the order of 10^−14^ m^2^W^−1^ ^40,41^, and high power above the threshold, are uniquely versatile devices where the end function can be user-defined post-fabrication. They can be readily employed as standalone optical components for filtering, frequency-selective phase delay, and resonant amplification and in transmitter and receiver modules enabling wavelength-division multiplexing in free-space optical links in the mid-IR^54^. Furthermore, the inherently small gain recovery time of QCLs^55^ suggests the use of QC ring resonators as high-speed GHz modulators, in both intensity modulation and phase modulation modes depending on the operating point of the device. We envision the full potential of these devices to be realized in highly agile mid-IR active PICs incorporating multiple resonators, couplers, and detectors, where the function of each component can be reconfigured on the go. One example of such integrated circuit is an on-chip wavelength-tunable external cavity laser, where one mirror of a linear Fabry-Perot cavity is replaced with a Vernier ring feedback element^56–58^. In such devices, modulation of the refractive index in the feedback element at MHz rate may enable frequency-swept spectrometers on par or surpassing the microsecond temporal resolution of state-of-the-art commercial mid-IR dual-comb spectrometers^59^. The benefit of purely active photonic integration is that the fabrication complexity does not scale with the number of integrated components as they are all defined in a single processing step and that the fabrication protocols are the ones that are already used at the industrial laser foundries. The downside of such approach is the necessity to apply electrical bias to each component even when its function is purely passive, such as light routing. A possible workaround to enable low-loss light guiding, although a more elaborate in fabrication, is the monolithic integration of QC active regions with passive waveguides by epitaxial regrowth in a III-V platform^23,26,27^.

When it comes to the control of the frequency comb states generated by ring QCLs above threshold, active directional couplers provide a new degree of freedom. To date, state control in QCL frequency combs is an unresolved issue. Existing approaches to laser state manipulation — engineering of the gain material and of cavity dispersion, mode selection — offer ad hoc laser state generation that is static and oftentimes unpredictable^39,60^. The external drive laser (which can be a separate unit as in this work or can straightforwardly be integrated on one laser chip) is a powerful control knob. According to the recent theoretical work, bringing the drive laser frequency in the vicinity of the frequency of the ring laser field (less than one FSR) and scanning through the lasing resonance should enable on-demand generation of bright and dark cavity solitons and Turing rolls, allowing for deterministic coherent control of the dynamical states, in analogy with passive Kerr microresonators^61^. Such driving schemes, in conjunction with an ease of integration of multiple active components (drive laser, ring resonator, directional coupler), may bring into reach electrically-driven chip-scale femtosecond mid-IR pulse generators with peak power levels in the 10–100 Watt range.

Concerning free-running frequency comb generation (in absence of the external control laser) state control can be enhanced by actively tunable dispersion through the control over mode hybridization between two strongly coupled resonators, as shown here on an example of RT and WG resonators^62^. Furthermore, a combination of an actively tunable coupled resonator system and a source of coherent radiation that encompasses in itself a giant optical nonlinearity — all in one, integrated electrooptic device — creates a versatile testbed for resonant electromagnetic phenomena, such as strong coupling and parity-time symmetry, in a new setting, where internally generated coherent multimode light can interact with a tunable potential created by the coupled resonators^37^. The demonstrated design, fabrication, and characterization methods are directly applicable to the implementation of a system of two-dimensional ring resonator arrays, where each resonator can be individually addressed via electrical pumping. When operated above the threshold, such system may provide a rich nonlinear optical playground for the studies of coherent interaction of the frequency comb states, their synchronization, and topological effects^63^.

## Methods

### Device fabrication and operation

The lasers emit at around 8.2 μm and have a structure consisting of GaInAs/AlInAs layers on an InP substrate. The active region consists of AlInAs/GaInAs/InP layers and the band structure design is based on a single-phonon continuum depopulation scheme. The waveguides are etched using the standard fabrication recipe using optical lithography. Waveguide width is 10 μm, the curved section of the racetrack is a semicircle with a radius of 500 μm, and the length of the straight section is 785 μm. The length of the waveguide coupler is 2.7 mm. The FSR is 18.6 GHz. The devices are driven at voltage levels between 7 V and 9 V and drive current levels between 100 mA and 500 mA.

### Transmission measurement setup

As a probe laser we employ a FP QCL fabricated from the same epitaxial material as the RT resonators. Even though FP QCLs tend to operate in multimode regime when driven well above the lasing threshold, we identified several devices that exhibited single-mode behavior over a large current range. To tune the probe wavelength we ramp the injection current of the FP QCL. We inject the light into the WG from the probe laser by focusing it onto a facet of the WG with an aspheric antireflection coated lens (NA = 0.56). Light transmitted through the WG is collected at the opposite facet with an identical lens and focused onto an HgCdTe infrared photodetector (Fig. 2a). To eliminate detection of the amplified spontaneous emission from the WG and to increase the signal-to-noise ratio we use lock-in detection (Zurich Instruments UHFLI 600 MHz lock-in amplifier) by chopping the probe beam at a rate of 320 Hz and demodulating the detector signal at the same frequency. All devices are operated under DC electrical bias with a low-noise current driver (Wavelength Electronics QCL LAB 1500 or QCL LAB 2000), and their temperature is stabilized at 16 °C using a low-thermal-drift temperature controller (Wavelength Electronics TC5).

### Resonance fitting for the extraction of the quality factor

Resonances of the coupled RT-WG system are well-described by the Fano resonance. First introduced by Ugo Fano is 1961, the Fano resonance is used to model interference of a discrete state with a continuum of states, leading to a distinct asymmetric profile^64^. The Fano resonance also shows up in photonics when two coupled oscillators with very different quality factors interact. In the case of the RT-WG system, the RT acts as a high quality resonator, while the WG acts as a lossy, low quality resonator. In general, the Fano resonance lineshape is given by^64^:

3 $$\sigma=\frac{{\left(\frac{2(\omega -{\omega }_{0})}{\Gamma }+q\right)}^{2}}{{\left(\frac{2(\omega -{\omega }_{0})}{\Gamma }\right)}^{2}+1},$$

where *ω*_0_ is the resonant frequency, Γ is a the resonance linewidth, and *q* is the so-called Fano parameter, which determines the direction and degree of asymmetry of the resonance profile. In the limit that *q* ⟶ 0, the Fano resonance profile becomes a classical Lorentzian “dip” profile, while when *q* ⟶ ± *∞* the Fano resonance profile becomes a Lorentzian “peak” profile. By increasing the bias of the RT, we increase its quality factor, which ultimately sweeps the Fano parameter for the resonances.

### Supplementary information

Supplementary Information

Peer Review File

## Data Availability

The experimental and numerical data generated in this study have been deposited in the Harvard Dataverse database and is available under this link. This data are made available under the Creative Commons Attribution 4.0 International License (CC BY 4.0).
