# Supplementary material for: Active mid-infrared ring resonators
Source: Nat Commun. 2024 Jan 19;15:607. doi: 10.1038/s41467-023-44628-7 (PMC10799048; doi:10.1038/s41467-023-44628-7)
Supplement: Supplementary file 1 — Supplementary Information [file 41467_2023_44628_MOESM1_ESM.pdf]

Supplementary Information to: Active mid-infrared ring resonators

Dmitry Kazakov,[1,*] Theodore P. Letsou,[1,2] Maximilian Beiser,[3] Yiyang Zhi,[1,4] Nikola Opačak,[1,3] Marco Piccardo,[1,5,6] Benedikt Schwarz,[1,3] and Federico Capasso[1,†]

[1]*Harvard John A. Paulson School of Engineering and Applied Sciences, Harvard University, Cambridge, MA 02138, USA*
[2]*Department of Electrical Engineering and Computer Science, Massachusetts Institute of Technology, Cambridge, MA 02142, USA*
[3]*Institute of Solid State Electronics, TU Wien, 1040 Vienna, Austria*
[4]*UC Berkeley, Berkeley, CA 94720, USA*
[5]*Department of Physics, Instituto Superior Técnico, Universidade de Lisboa, 1049-001 Lisbon, Portugal*
[6]*Instituto de Engenharia de Sistemas e Computadores – Microsistemas e Nanotecnologias (INESC MN), 1000-029 Lisbon, Portugal*

I. DIRECTIONAL COUPLER DESIGN

To optimize the coupler geometry, ultimately constrained by the resolution of the optical lithography, we first found the indices of the even (n_e) and odd (n_o) eigenmodes of the coupled waveguide structure using COMSOL (Fig. S1**a**, **b**). Initially distinct values of n_e and n_o for closely spaced, strongly coupled waveguides, approach the same value as the waveguides get further apart (Fig. S1**c**). The difference between n_e and n_o also gets more pronounced the narrower the waveguides are (Fig. S1**d**). The contrast between the even and odd mode indices $k = n_e - n_o$ and the length of the interaction region L_{int} then define the power coupling, for the wavelength $\lambda = 7.9$ µm, according to

$$|\kappa|^2 = \frac{1}{1+(\delta/k)^2}\sin^2\left(\frac{\pi\sqrt{k^2+\delta^2}}{\lambda}L_{\mathrm{int}}\right), \tag{S1}$$

where $\delta = n_{\mathrm{RT}} - n_{\mathrm{WG}}$ is the mode index mismatch in the coupled waveguides, that is set to zero in the simulation, but in the experiment may increase as we bias the WG and RT independently. Coupling coefficient can thus be increased by decreasing the separation between the waveguides (Fig. S1**e**), decreasing the waveguide width (Fig. S1**f**), and by increasing the length of the interaction region (Fig. S1**e**, **f**). The system will become overcoupled for too large L_{int} or for very narrowly spaced waveguides (Fig. S2).

II. CIRCULAR RING QCLS WITH ACTIVE COUPLERS

Here we show that circular ring QCLs can as well support an integration of a directional coupler. Here the coupler is implemented as a segment of a ring of a larger radius, concentric with the ring laser cavity (Fig. S3**a**). We fabricated and tested such devices (Fig. S3**b**), which behave similarly to RT QCLs of the main text. In this geometry, as opposed to the coupling section with two straight waveguide segments, an additional reduction in coupling may come from the mode index mismatch in the two waveguides with different radii of curvature. The lower is the radius of curvature the more the mode gets pushed away from the center of the waveguide towards the outer rim of the ring, which results in higher mode index (Fig. S3**c**, **e**). This effect gets more pronounced for wider waveguides (Fig. S3**d**, **f**). Similarly to the straight coupler geometry, the power coupling between two curved waveguides coefficient decreases with increasing gap width (Fig. S3**g**).

III. CAVITY TRANSMISSION MODEL AND FITTING

We model and fit the RT-WG system below threshold using the transfer matrix method in order to extract various parameters of the system, notably the refractive index, coupling coefficient, and refractive index change due to thermal

---

* kazakov@seas.harvard.edu
† capasso@seas.harvard.edu

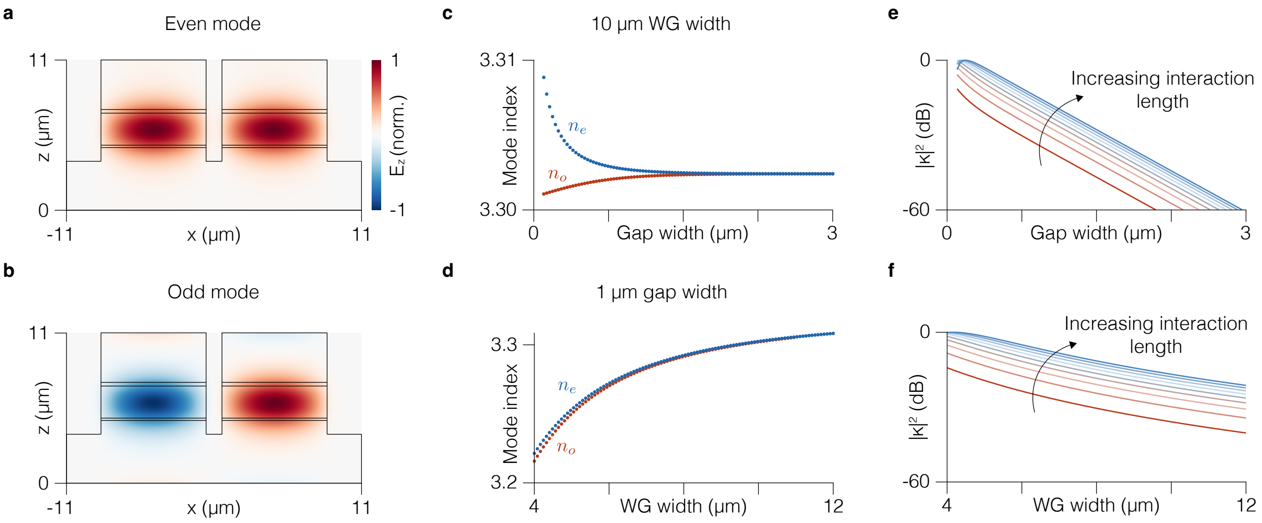

FIG. S1. **a**, Simulated component of the electric field of the even mode oriented in direction of epitaxial growth. **b**, Simulated component of the electric field of the odd mode oriented in direction of epitaxial growth. **c**, Simulated even and odd mode indices as function of the width of the gap between the waveguides. **d**, Simulated even and odd mode indices as function of the waveguide width. **e**, Computed power coupling coefficient $|\kappa|^2$ as function of the gap width for the waveguide width of 10 μm. **f**, Computed power coupling coefficient $|\kappa|^2$ as function of the waveguide width for the gap width of 1 μm.

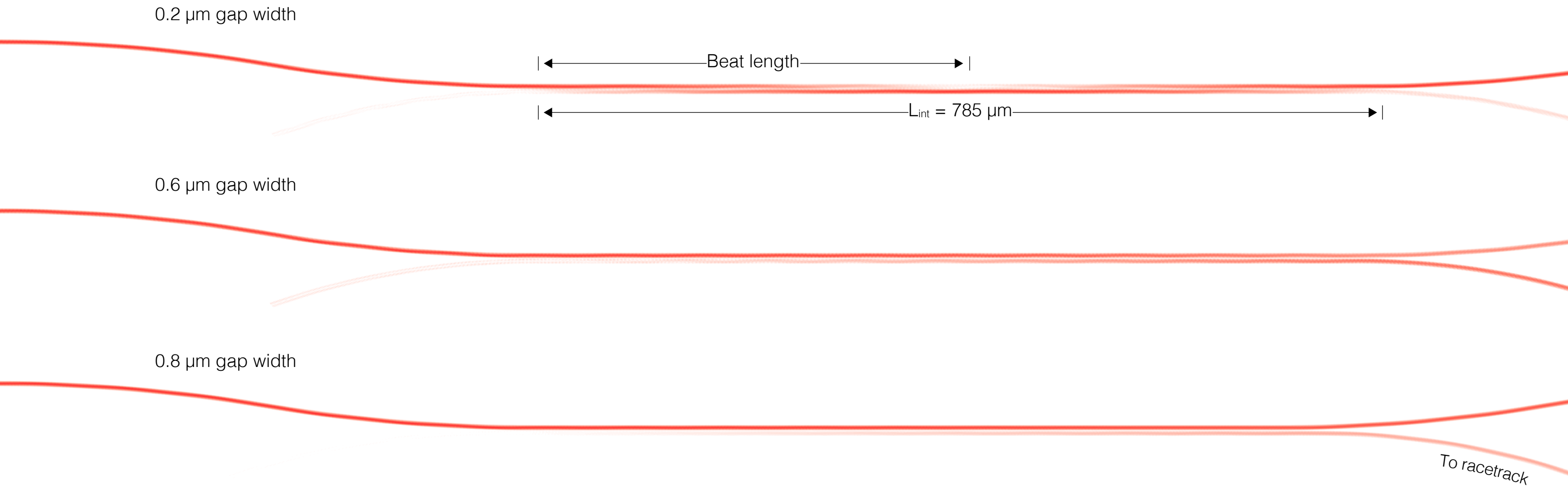

FIG. S2. 2D COMSOL simulation of field intensity in a waveguide coupled to the racetrack for three different values of the gap width.

crosstalk between the WG and RT. The transfer matrix, M, is a 2×2 matrix that relates the counter-propagating input field components, a_{in} and b_{in}, to the output field components a_{out} and b_{out} via

$$\begin{pmatrix} a_{\text{out}} \\ b_{\text{out}} \end{pmatrix} = M \begin{pmatrix} a_{\text{in}} \\ b_{\text{in}} \end{pmatrix} \tag{S2}$$

M, is the product of all substituent transfer matrices that represent individual interactions in the system, $M = M_{\text{F}} M_{\text{WG}} M_{\text{RT}} M_{\text{WG}} M_{\text{F}}$. Here, M_{F} represents interface scattering from either air to WG or WG to air, M_{WG} represents propagation along half of the WG, and M_{RT} represents coupling to and propagation of the mode in the RT resonator.

Assuming propagation along the $+x$ direction using the time convention of $\exp\{i(\omega t - \beta x)\}$, where ω is the angular

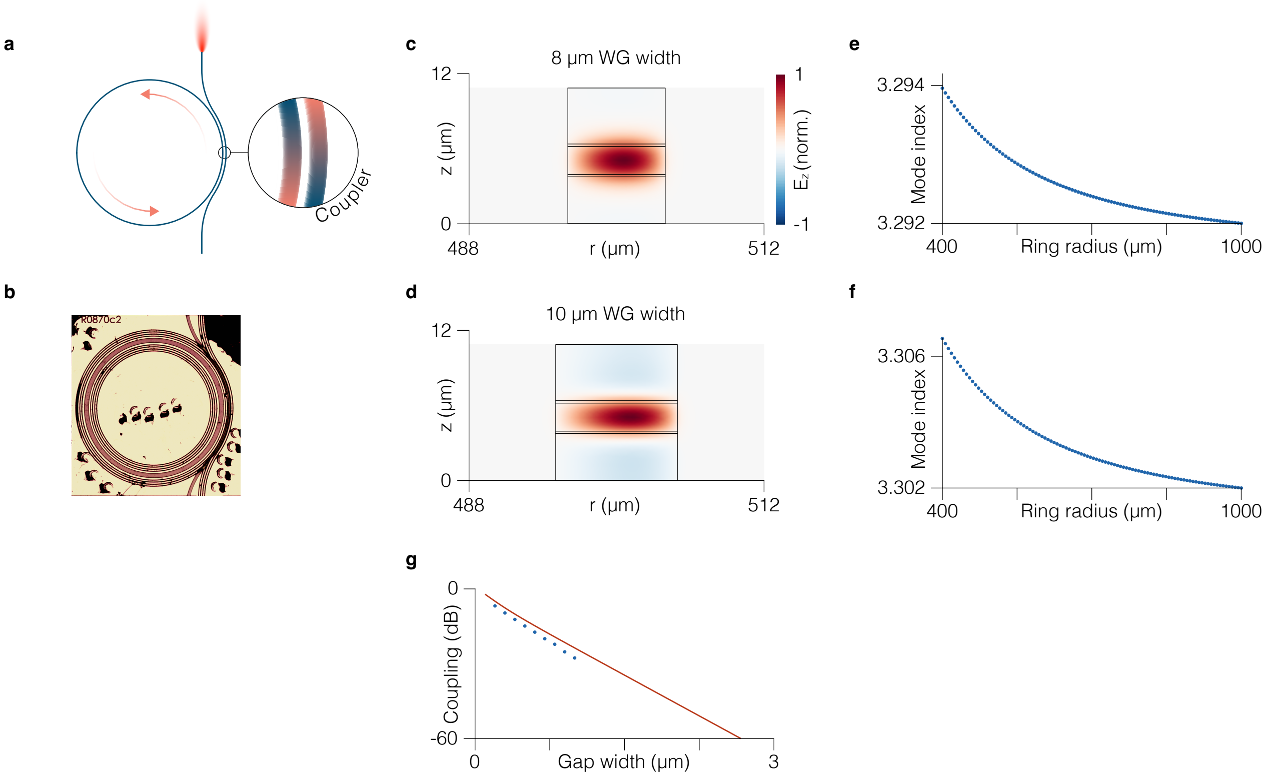

FIG. S3. **a**, Circular ring QCL with a bent waveguide coupler implemented as a segment of a ring of a larger radius, concentric with the ring QCL. **b**, Optical micrograph of a fabricated circular ring QCL with a waveguide coupler. **c**, Simulated z-component (in cylindrical coordinates) of the electric field of the fundamental mode of the 8 µm wide waveguide of a circular ring with a radius of 500 µm. **d**, Same as in **c**, but for a waveguide width of 10 µm. The mode gets noticeably pushed to the outer boundary of the ring waveguide. **e**, Simulated mode index in a 8 µm wide circular ring waveguide as a function of the ring radius. **f**, same as in **e**, but for a waveguide width of 10 µm. **g**, Computed (solid line) and simulated (dots) power coupling coefficient for a circular ring as a function of gap width.

frequency of the injected field and β is the propagation constant, the individual transfer matrices are given by:

$$M_{\mathrm{F}} = \frac{1}{2n_2}\begin{pmatrix} n_2+n_1 & n_2-n_1 \\ n_2-n_1 & n_2+n_1 \end{pmatrix}, \tag{S3}$$

$$M_{\mathrm{WG}} = \begin{pmatrix} \exp\left(-i\beta_{\mathrm{WG}}L_{\mathrm{WG}}/2\right) & 0 \\ 0 & \exp\left(i\beta_{\mathrm{WG}}L_{\mathrm{WG}}/2\right) \end{pmatrix}, \tag{S4}$$

$$M_{\mathrm{RT}} = \begin{pmatrix} t_{\mathrm{RT}} & 0 \\ 0 & 1/t_{\mathrm{RT}} \end{pmatrix}, \tag{S5}$$

$$t_{\mathrm{RT}} = \frac{\sqrt{1-\kappa^2} - \exp\left(-i\beta_{\mathrm{RT}}C_{\mathrm{RT}}\right)}{1-\sqrt{1-\kappa^2}\exp\left(-i\beta_{\mathrm{RT}}C_{\mathrm{RT}}\right)}. \tag{S6}$$

In Eq. S3, n_1 and n_2 are the (generally) complex mode indices of the two media on the opposite sides of the interface (air and the waveguide). In Eq. S4, $\beta_{\mathrm{WG}} = \frac{2\pi n_{\mathrm{WG}}}{\lambda}$ is the propagation constant of the WG, where λ is wavelength of the injected field, and L_{WG} is the length of the WG. In Eq. S6, κ is the amplitude coupling coefficient, that can be computed from Eq. S1, β_{RT} is the propagation constant of the RT, and C_{RT} is the circumference of the RT. Assuming no initial counter-propagation at the output ($b_{\mathrm{out}} = 0$), the complex transmission coefficient is given by:

$$\frac{a_{\mathrm{out}}}{a_{\mathrm{in}}} = \frac{1}{M(2,2)}\left(M(1,1)M(2,2) - M(1,2)M(2,1)\right). \tag{S7}$$

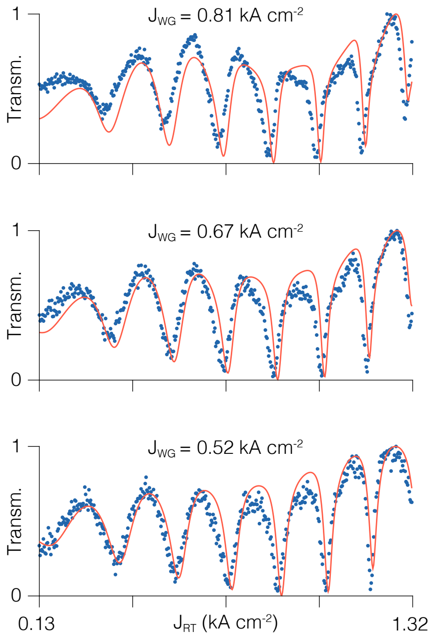

FIG. S4. Optimized fitting (red) of Eq. S7 on three experimentally-obtained transmission spectra (blue), corresponding to $J_{\mathrm{WG}} = 0.524$ kA/cm^2, $J_{\mathrm{WG}} = 0.668$ kA/cm^2, $J_{\mathrm{WG}} = 0.813$ kA/cm^2.

Eq. S7 is used to fit the experimental transmission of the RT-WG system shown in the main text.

In order to accurately reproduce the experimental data, we assume the refractive index of either the WG or RT changes as a function of current density, J, to an arbitrary power:

$$n_{\mathrm{RT}}(J_{\mathrm{RT}}) = n_{\mathrm{RT}_0} + \Delta n_{\mathrm{RT}} J_{\mathrm{RT}}^{\alpha}, \tag{S8}$$

$$n_{\mathrm{WG}}(J_{\mathrm{WG}}) = n_{\mathrm{WG}_0} + \Delta n_{\mathrm{WG}} J_{\mathrm{WG}}^{\alpha}, \tag{S9}$$

where $n_{\mathrm{RT,WG}_0}$ is the base refractive index and $\Delta n_{\mathrm{RT,WG}}$ is how much the index changes per unit J^{α}. We also model crosstalk between the RT and WG, i.e. how heating of either the RT or WG due to biasing affects the refractive index of the other section. It is accounted for by additional contribution, $\Delta n_{\mathrm{cross}}$, to the refractive index of either the RT or WG, which is directly proportional the current density of the other section:

$$n_{\mathrm{RT}}(J_{\mathrm{RT}}, J_{\mathrm{WG}}) = n_{\mathrm{RT}}(J_{\mathrm{RT}}) + \Delta n_{\mathrm{cross}} J_{\mathrm{WG}} \tag{S10}$$

$$n_{\mathrm{WG}}(J_{\mathrm{WG}}, J_{\mathrm{RT}}) = n_{\mathrm{WG}}(J_{\mathrm{WG}}) + \Delta n_{\mathrm{cross}} J_{\mathrm{RT}}. \tag{S11}$$

We select three experimentally-measured transmission spectra (corresponding to $J_{\mathrm{WG}} = 0.524$ kA/cm^2, $J_{\mathrm{WG}} = 0.668$ kA/cm^2, $J_{\mathrm{WG}} = 0.813$ kA/cm^2) to perform least squares fitting using Eq. S7, as shown in Fig. S4.

IV. QUALITY FACTOR DEFINITION

The quality factor of the resonance is defined as $Q = k/\delta k$, where $k = 2\pi/\lambda$ and δk (full width at half minimum) is derived from resonator transmission formula in the main text and reads as follows:

$$\delta k(\alpha, t) = 2 \cdot \arccos\left(\frac{\frac{\alpha^2+t^2-2\alpha t}{1+\alpha^2 t^2+2\alpha t} + \frac{\alpha^2+t^2+2\alpha t}{1+\alpha^2 t^2-2\alpha t}}{2\alpha t\left(\frac{\alpha^2+t^2-2\alpha t}{1+\alpha^2 t^2+2\alpha t} + \frac{\alpha^2+t^2+2\alpha t}{1+\alpha^2 t^2-2\alpha t} - 2\right)}\right) \tag{S12}$$

V. COHERENCE CHARACTERIZATION OF THE COMB STATE

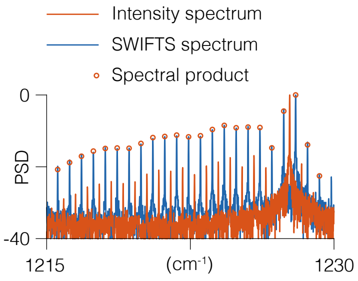

FIG. S5. **Phase coherence of the comb spectrum.** Intensity spectrum and SWIFTS spectrum of a RT QCL above the threshold. The geometric means of the neighboring mode pairs (spectral product) overlap with the peaks in the SWIFTS spectrum, indicating coherence of the state across entire spectral bandwidth. FSR of the resonator is 18.6 GHz.

While a narrow intermode beat note is an indication of the comb coherence, it is not a rigorous proof thereof, as some of the lines in the multimode spectrum may be locked separately from the rest or remain unlocked, thus not contributing to the intermode beat note seen in the RF spectrum centered at the resonator roundtrip frequency. The technique that allows to bypass this limitation is known as SWIFTS and relies on discriminating the pairs of modes in the comb spectrum and measuring the phase and the amplitude of the intermode beat note coming from each pair separately. The most convenient implementation of this technique involves a fast linear detector at the output of a Michelson interferometer, from which intensity components at DC and at the intermode beat frequency are obtained as a function of optical delay, resulting in a linear DC interferogram and a SWIFTS interferogram. SWIFTS interferogram is complex and contains an in-phase and a quadrature components, that are obtained by passing the output of the detector output through a lock-in amplifier. The magnitude of the Fourier transform of the SWIFTS interferogram for a representative frequency comb state is shown in Fig. S5. The complete coherence of the comb lines over the spectral span is confirmed by a near-perfect overlap of the calculated pairwise geometric means between the comb teeth in the intensity spectrum (spectral product, red circles) and the maxima of the peaks in the SWIFTS spectrum.

VI. SPECTRAL CHARACTERIZATION OF MORE DEVICES

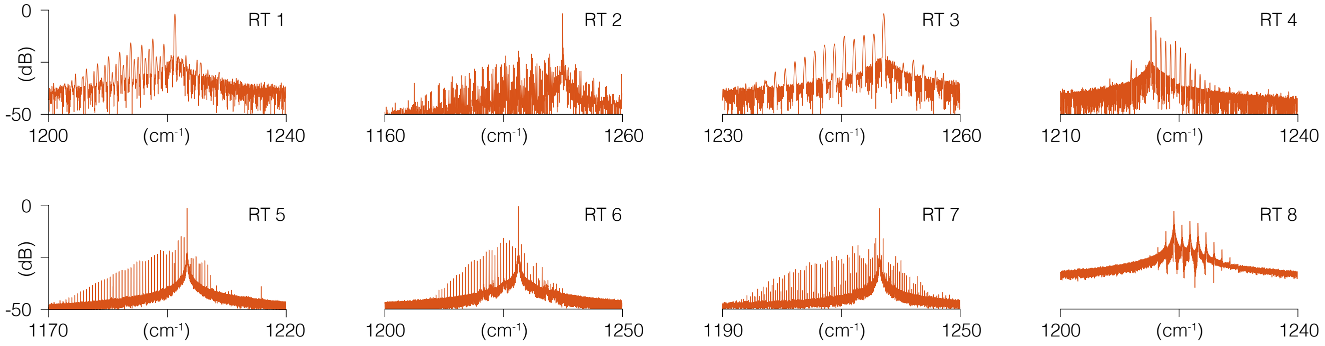

FIG. S6. **Optical spectra of eight representative RT QCLs with directional couplers.**

To demonstrate the universality of the phenomenon of comb formation in RT QCLs we recorded the emission spectra of eight more devices. All RTs generate frequency combs (Fig. S6), similarly to the device in the main text.

VII. FUTURE DEVICES BASED ON ACTIVE MID-INFRARED RING RESONATORS

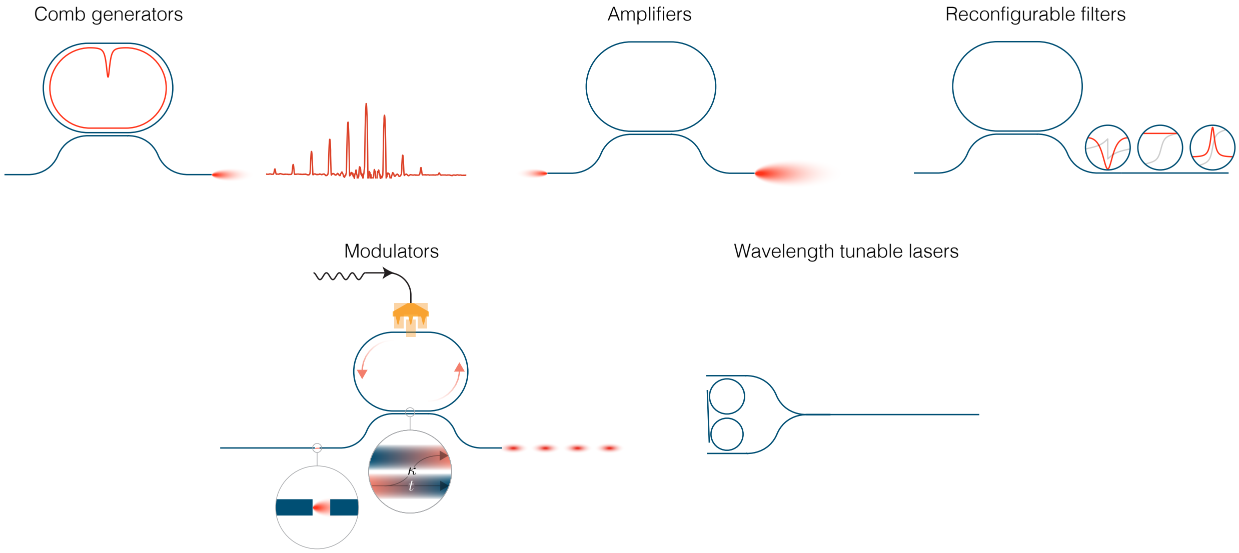

FIG. S7. **Future device architectures based on active mid-IR ring resonators shown in the main text.**

Active ring resonators will enable a plethora of devices in the future, all stemming from the concepts demonstrated in the main text. Here we showcase a non-exhaustive library of such photonic integrated components (Fig. S7):

1. Frequency comb generators, based both on free-running ring QCLs and coherently driven resonators.
2. Resonant amplifiers based on active ring resonators driven above the transparency point.
3. Reconfigurable and tunable notch filters and resonant phase shifters, that can be used to manipulate the individual lines of the frequency combs generated by active ring resonators above the lasing threshold.
4. Fast (GHz) resonant modulators.
5. Chip-scale wavelength tunable lasers where the active ring resonators operated below threshold will serve as feedback elements in a hybrid cavity together with a semiconductor optical amplifier (a QCL waveguide biased above the transparency).

Despite being long available in the near-IR, most of these integrated components have not yet been developed in the mid-IR range.
