# Supplementary material for: Active mid-infrared ring resonators
Source: Nat Commun. 2024 Jan 19;15:607. doi: 10.1038/s41467-023-44628-7 (PMC10799048; doi:10.1038/s41467-023-44628-7)
Supplement: Supplementary file 2 — Peer Review File [file 41467_2023_44628_MOESM2_ESM.pdf]

nature portfolio

Peer Review File

Active mid-infrared ring resonators

Open Access This file is licensed under a Creative Commons Attribution 4.0 International License, which permits use, sharing, adaptation, distribution and reproduction in any medium or format, as long as you give appropriate credit to the original author(s) and the source, provide a link to the Creative Commons license, and indicate if changes were made. In the cases where the authors are anonymous, such as is the case for the reports of anonymous peer reviewers, author attribution should be to 'Anonymous Referee' followed by a clear attribution to the source work. The images or other third party material in this file are included in the article's Creative Commons license, unless indicated otherwise in a credit line to the material. If material is not included in the article's Creative Commons license and your intended use is not permitted by statutory regulation or exceeds the permitted use, you will need to obtain permission directly from the copyright holder. To view a copy of this license, visit http://creativecommons.org/licenses/by/4.0/.

REVIEWER COMMENTS

Reviewer #1 (Remarks to the Author):

In the manuscript, "Active mid-infrared resonators," Dmitry Kazakov and collaborators demonstrate the integration of directional couplers and mid-infrared resonators that incorporate mid-infrared quantum cascade active regions in the waveguide core. The authors show how this integration can be leveraged for tunable filters, nonlinear frequency conversion, and frequency comb generation—all from a single device by changing the way that the device is driving. Overall, this is an extremely well written paper with very high presentation quality. My recommendation is that the manuscript be published in Nature Communications after the authors have addressed some minor comments below. I do not see any of these comments as mandatory changes, but I do think that addressing them will improve the clarity of the paper.

1. Following equation (1), the authors discuss the parameter \alpha. I believe there is an error/typo when discussing this parameter. In lines 73 and 74, the authors indicate that n''>0 for absorption. In the definition of \alpha, for any L > \lambda, n''>0 results in \alpha > 1. The authors state in line 82, that \alpha >1 indicates gain. It seems that the definition of "absorption" and "gain" in lines 73 and 74 is flipped.
2. It would be useful to the reader for the authors to directly link the quality factor to the other quantities that they have defined. This would be particularly helpful in the section, "Regime control and tunability of active QC resonators."
3. In Fig. 2b, the authors label two portions of the figure as "weak coupling" and "strong coupling". In the main text, (Page 6, lines 160 – 162), the authors discuss the strong coupling and the avoided crossing seen in the experimental data. I do agree that "weak" and "strong" regimes look different, but it's not clear how to extend these differences immediately to weakly and strongly coupled resonators. Typically, wavelength-dependent measurements are used to demonstrate avoided crossings (or lack thereof). The reference the authors cite employs wavelength-dependent measurements. It's not clear how the data the authors present (fixed wavelength, sweeping J_RT and J_WG) is related to the more common observations of avoided crossings.
4. How do the authors know that the lasing direction in the RT is unidirectional? My understanding is that the lasing direction is often bidirectional? See for example, "Unidirectional mode selection in bistable quantum cascade ring lasers." How important is the claim? Can it be substantiated in some way?
5. It might be useful to include an optical spectra for other detunings. This helps establish that the mixing is occurring in the RT and not the WG. I believe it should be possible to have mixing in the WG since the RT and WG share the same active region.

Reviewer #2 (Remarks to the Author):

The manuscript by the Capasso group presents the development and implementation of mid-infrared ring resonators and directional couplers with a quantum cascade laser (QCL) active region. The design allows for electrical control of various parameters such as resonant frequency, quality factor, coupling regime, and the coupling coefficient. Depending on its operating point, the device is purported to act as a tunable filter, a nonlinear frequency converter, or a frequency comb generator, which the authors experimentally and theoretically characterize.

While the work is interesting, in my opinion it is not sufficiently original and is not adequately suited to the broad, interdisciplinary audience of Nature Communications. Ring resonators and directional couplers have been integrated into active waveguides for over two decades (Optics Letters 26, 506–508 (2001), IEEE Journal of Quantum Electronics 39, 859–865 (2003), Optics Express 23, 3221–3229 (2015)), and all of the elements demonstrated here have been

demonstrated in the near-infrared. The main novelty in this work is the fact that it was done in the mid-infrared using QCLs. While QCLs do have some differences from diode lasers, especially with respect to their gain recovery dynamics, that fact is not especially relevant to this work.

The importance of this work is more technical and is more germane to an audience with specialized interests. Therefore, I suggest that a more specialized journal, such as Optics Letters or Applied Physics Letters, would be more appropriate for this manuscript. This would ensure that the research is disseminated amongst a readership that can fully appreciate and utilize the technical results of this work.

Though I could not support publication in Nature Communications, for informational purposes I would suggest that the authors address the following questions and comments to improve their manuscript:
a. The claims of nonlinear frequency conversion are not proven. The peak that is alleged to be an idler is orders of magnitude lower than the pump & signal and were measured using FTIR, which means that a large range of nonlinear artifacts could create the same effect: detector saturation, quantization artifacts, etc. Additiionally, the leftmost spectrogram in Fig. 3b seems to show that the peak is present over the whole detuning range, which would indicate that it is not dependent on coupling into the ring. To conclusively establish that the peak alleged to be an idler is real, the authors should spectrally filter out the pump and signal, e.g. by using a grating as in Applied Physics Letters 102, 222104 (2013).
b. Are the high-frequency fluctuations evident in the detuning plots of Fig. 2a noise or actual signal? If they are noise, is there any particular reason that the SNR of these measurements is relatively low? Generally one would expect higher SNR with a powerful QCL and a HgCdTe detector.
c. Is the spectrum shown in Fig. 4c truly a comb, or merely comb-like?
d. In my opinion, the first paragraph of the introduction is extremely misleading, as it is incredibly unlikely that this result has any relevance to data centers or to optical signal processors. There is essentially no reason to do interconnects in the mid-infrared. Basically every aspect of the chain is worse: the detectors are noisier due to lower hv/kT, the lasers are more expensive to manufacture and have lower efficiency, the fibers have higher losses and are more expensive, etc. This paragraph reads like an attempt to entice a general audience, but it does so by misleading them.

Reviewer #3 (Remarks to the Author):

The Authors present a solid experimental study on active mid-infrared racetrack resonators based on quantum-cascade material. The layout of the proposed device is surely not new since many of these devices have been widely investigated lately in integrated optics with PIC.s. The novelty is that here the material of both racetrack and waveguide is active, allowing for a dynamical change of the coupling characteristics by acting on the pumping of the different elements.

The investigation is well conducted, and the results are surely interesting, even though I am not completely convinced they are fully matching the required general interest for Nature Comms. audience. There are some points that I would like the Authors to clarify/change.

1. In terms of references, in the QCL community there have been already a few implementations of PICs. Particularly, one experiment that is very similar to the one presented is described in the paper from Kundu, I. et al. Terahertz photonic integrated circuit for frequency tuning and power modulation. Opt. Express 28, 4374–4386. (2020). I would suggest to cite also this work.

2. The paper lacks a few quantitative informations: since it is integrated optics, it would be useful to know the actual value of current and applied bias (and not only current density) to evaluate the power load of the proposed device, both for Racetrack and Waveguide. Also the FSR of the

racetrack and the coupler waveguide is missing from the paper.

3. Regarding the comb operation, there is little discussion about the actual coherence of the modes presented in Fig 4 c. Ideally it would be necessary to show SWIFTs or dual-comb signals to actually prove the comb nature of the laser emission. In this case, since there is an analysis of the symmetry breaking and a measurement of the CW and CCW light emission, it would be sufficient to show an optical or electrical beatnote (should be around 15 GHz so not so high in frequency...quite easy to measure.). I would say that a beat note measurement is a necessary requirement to affirm that the RT is producing a comb state.

4. Still on the fig4c. is the envelope of the modes sech^2 ? otherwise is really not clear why this should be a comb

In conclusion, the Authors should produce more convincing data to substantiate the claim of comb operation.

- Reviewer comments are in red, shown without editing, as they appeared in the original communication from the editor
- Our responses are in blue
- Summaries of the implemented changes are in green

REVIEWER COMMENTS

Reviewer #1 (Remarks to the Author):

In the manuscript, “Active mid-infrared resonators,” Dmitry Kazakov and collaborators demonstrate the integration of directional couplers and mid-infrared resonators that incorporate mid-infrared quantum cascade active regions in the waveguide core. The authors show how this integration can be leveraged for tunable filters, nonlinear frequency conversion, and frequency comb generation—all from a single device by changing the way that the device is driving. Overall, this is an extremely well written paper with very high presentation quality. My recommendation is that the manuscript be published in Nature Communications after the authors have addressed some minor comments below. I do not see any of these comments as mandatory changes, but I do think that addressing them will improve the clarity of the paper.

We thank Reviewer #1 for the positive evaluation of the work, recommendation to publish in Nature Communications, and for additional remarks that helped to further improve our manuscript.

1. Following equation (1), the authors discuss the parameter \alpha. I believe there is an error/typo when discussing this parameter. In lines 73 and 74, the authors indicate that n’’>0 for absorption. In the definition of \alpha, for any L > \lambda, n’’>0 results in \alpha > 1. The authors state in line 82, that \alpha >1 indicates gain. It seems that the definition of “absorption” and “gain” in lines 73 and 74 is flipped.

We corrected the sign in the exponent in the formula for \alpha in line 83.

2. It would be useful to the reader for the authors to directly link the quality factor to the other quantities that they have defined. This would be particularly helpful in the section, “Regime control and tunability of active QC resonators.”

We now provide the mathematical definition of the quality factor in the Supplementary Section IV.

3. In Fig. 2b, the authors label two portions of the figure as “weak coupling” and “strong coupling”. In the main text, (Page 6, lines 160 – 162), the authors discuss the strong coupling and the avoided crossing seen in the experimental data. I do agree that “weak” and “strong” regimes look different, but it’s not clear how to extend these differences immediately to weakly and strongly coupled resonators. Typically, wavelength-

dependent measurements are used to demonstrate avoided crossings (or lack thereof). The reference the authors cite employs wavelength-dependent measurements. It's not clear how the data the authors present (fixed wavelength, sweeping J_RT and J_WG) is related to the more common observations of avoided crossings.

Following definition of Eq. 1, the resonance in transmission can be observed in two ways: either sweeping the wavenumber of the probe laser ($k = 2\pi/\lambda$) or the refractive index of the waveguide n'. Sweeping either of these parameters results in an equivalent effect — a resonant dip in transmission. In an experiment we see resonances when sweeping the electrical current of the racetrack (RT) and waveguide (WG) (Fig. 2b), as well as sweeping the wavenumber of the probe laser (Fig. 2a).

The same equivalence principle holds in the case of the coupled resonator system. In our experiments we choose to sweep the bias (and therefore refractive indices) of the resonators due to the limited wavelength coverage range of the probe laser, that would not allow to continuously scan across the coupled resonance, as the resonance itself would move with changing currents of the WG and the RT (again, due to index change). We therefore chose to fix the wavelength of the probe laser and perform the scan of the refractive index of the resonators instead, to be able to cover the wide range of coupling conditions and resonator quality factors.

To address this comment of Reviewer #1, we appended the sentence that starts at line 90 of the main text as follows:

The detuning of the input field from the cavity resonance--that enters Eq.~\ref{eq:Yariv} through the cosine term--is controlled via the real part of the refractive index (equivalently, the detuning from the resonance can be controlled by changing the wavenumber $k=2\pi/\lambda$).

4. How do the authors know that the lasing direction in the RT is unidirectional? My understanding is that the lasing direction is often bidirectional? See for example, "Unidirectional mode selection in bistable quantum cascade ring lasers." How important is the claim? Can it be substantiated in some way?

Unidirectional operation is evident from the measurement of the output intensity from both ports of the WG coupler, as shown in Fig. 4b. Right above the threshold the laser is bidirectional and becomes unidirectional at higher bias as a result of mode competition between clockwise (CW) and counterclockwise (CCW) waves.

The same type of measurement is performed in the reference cited by Referee #1 (Ref. 51 of the main text), with the difference being in that work the authors use pulsed biasing, and mode selection (CW or CCW) is influenced by the length and the duty cycle of the bias pulses. In our case, the laser behaves well in accordance with the theory of a two-mode laser in absence of a mode selection mechanism.

5. It might be useful to include an optical spectra for other detunings. This helps

establish that the mixing is occurring in the RT and not the WG. I believe it should be possible to have mixing in the WG since the RT and WG share the same active region.

Optical spectrograms with varying detuning are provided in Fig. 3b (top panels), where the cuts are taken along the detuning value where the idler sideband intensity is maximized. It is evident from the spectrogram that the idler sideband is above the noise floor only for a limited range of the detuning values when the signal wave is tuned into one of the resonances of the ring laser. It is exactly for this reason indicated by Reviewer #1 — to exclude the possibility of mixing inside the WG, which is an active nonlinear medium as well — why we performed this measurement.

Reviewer #2 (Remarks to the Author):

The manuscript by the Capasso group presents the development and implementation of mid-infrared ring resonators and directional couplers with a quantum cascade laser (QCL) active region. The design allows for electrical control of various parameters such as resonant frequency, quality factor, coupling regime, and the coupling coefficient. Depending on its operating point, the device is purported to act as a tunable filter, a nonlinear frequency converter, or a frequency comb generator, which the authors experimentally and theoretically characterize.

While the work is interesting, in my opinion it is not sufficiently original and is not adequately suited to the broad, interdisciplinary audience of Nature Communications. Ring resonators and directional couplers have been integrated into active waveguides for over two decades (Optics Letters 26, 506–508 (2001), IEEE Journal of Quantum Electronics 39, 859–865 (2003), Optics Express 23, 3221–3229 (2015)), and all of the elements demonstrated here have been demonstrated in the near-infrared. The main novelty in this work is the fact that it was done in the mid-infrared using QCLs. While QCLs do have some differences from diode lasers, especially with respect to their gain recovery dynamics, that fact is not especially relevant to this work.

The importance of this work is more technical and is more germane to an audience with specialized interests. Therefore, I suggest that a more specialized journal, such as Optics Letters or Applied Physics Letters, would be more appropriate for this manuscript. This would ensure that the research is disseminated amongst a readership that can fully appreciate and utilize the technical results of this work.

We appreciate the fact the referee finds our work interesting and appealing to a specialized audience. While we recognize that the demonstrated device — an active ring resonator with an integrated coupler — is not novel *conceptually*, we disagree on its limited potential impact or a lack of originality. It should not come as a surprise that significant technological advancements that stem from established concepts or ideas can still have a broad impact on science and technology. For example, the first waveguide-based ring resonator with directional coupler was reported back in 1980 (Haavisto, J. and Pajer, G.A., 1980. Resonance effects in low-loss ring waveguides. *Optics Letters*, *5*(12),

pp.510-512.), whereas devices *conceptually* identical to those, a few decades later, keep appearing in top scientific journals. Two recent examples are ring-based phase modulators in the visible range (Liang, G., Huang, H., Mohanty, A., Shin, M.C., Ji, X., Carter, M.J., Shrestha, S., Lipson, M. and Yu, N., 2021. Robust, efficient, micrometer-scale phase modulators at visible wavelengths. *Nature Photonics*, *15*(12), pp.908-913.) and wavelength-tunable external cavity lasers (Corato-Zanarella, M., Gil-Molina, A., Ji, X., Shin, M.C., Mohanty, A. and Lipson, M., 2023. Widely tunable and narrow-linewidth chip-scale lasers from near-ultraviolet to near-infrared wavelengths. *Nature Photonics*, *17*(2), pp.157-164.). In both works ring resonators are the enabling component to achieve key functions and represent a technological advancement rather than a *conceptually* new idea. Nevertheless, they are significant in that they extend existing concepts to new spectral regions. It is precisely what we do in our work — demonstrate active ring resonators with active waveguide couplers in a spectral region where such devices with functions described in our manuscript were not demonstrated previously. The significance of the mid-IR spectral region where our devices operate is widely recognized — among many others by Nature Communications as well. A quick search of the papers published in this journal carrying “mid-infrared” keyword in the title yields the following representative list of publications covering a broad scope of topics from chemistry, spectroscopy, astronomy, and imaging, to telecommunications, metasurfaces, detectors, and lasers:

1. Zhao, Y., Kusama, S., Furutani, Y., Huang, W.H., Luo, C.W. and Fuji, T., 2023. High-speed scanless entire bandwidth **mid-infrared chemical imaging**. *Nature Communications*, *14*(1), p.3929.
2. Wang, Y., Huang, K., Fang, J., Yan, M., Wu, E. and Zeng, H., 2023. **Mid-infrared single-pixel imaging** at the single-photon level. *Nature Communications*, *14*(1), p.1073.
3. Roberts, G., Ballew, C., Zheng, T., Garcia, J.C., Camayd-Muñoz, S., Hon, P.W. and Faraon, A., 2023. 3D-patterned inverse-designed **mid-infrared metaoptics**. *Nature Communications*, *14*(1), p.2768.
4. Zhu, J., Ji, S., Ren, Z., Wu, W., Zhang, Z., Ni, Z., Liu, L., Zhang, Z., Song, A. and Lee, C., 2023. Triboelectric-induced ion mobility for artificial intelligence-enhanced **mid-infrared gas spectroscopy**. *Nature Communications*, *14*(1), p.2524.
5. Wang, F., Hu, F., Dai, M., Zhu, S., Sun, F., Duan, R., Wang, C., Han, J., Deng, W., Chen, W. and Ye, M., 2023. A two-dimensional **mid-infrared optoelectronic retina** enabling simultaneous perception and encoding. *Nature Communications*, *14*(1), p.1938.
6. Liu, M., Gray, R.M., Costa, L., Markus, C.R., Roy, A. and Marandi, A., 2023. **Mid-infrared cross-comb spectroscopy**. *Nature Communications*, *14*(1), p.1044.
7. Zou, K., Pang, K., Song, H., Fan, J., Zhao, Z., Song, H., Zhang, R., Zhou, H., Minoofar, A., Liu, C. and Su, X., 2022. High-capacity **free-space optical communications** using wavelength-and mode-division-multiplexing in the mid-infrared region. *Nature Communications*, *13*(1), p.7662.

8. Ma, J., Cheng, B., Li, L., Fan, Z., Mu, H., Lai, J., Song, X., Yang, D., Cheng, J., Wang, Z. and Zeng, C., 2022. Unveiling Weyl-related optical responses in semiconducting tellurium by **mid-infrared circular photogalvanic effect**. *Nature Communications*, *13*(1), p.5425.
9. Hinkov, B., Pilat, F., Lux, L., Souza, P.L., David, M., Schwaighofer, A., Ristanić, D., Schwarz, B., Detz, H., Andrews, A.M. and Lendl, B., 2022. A **mid-infrared lab-on-a-chip** for dynamic reaction monitoring. *Nature communications*, *13*(1), p.4753.
10. Dai, M., Wang, C., Qiang, B., Wang, F., Ye, M., Han, S., Luo, Y. and Wang, Q.J., 2022. On-chip **mid-infrared photothermoelectric detectors** for full-Stokes detection. *Nature Communications*, *13*(1), p.4560.
11. Goldstein, J., Lin, H., Deckoff-Jones, S., Hempel, M., Lu, A.Y., Richardson, K.A., Palacios, T., Kong, J., Hu, J. and Englund, D., 2022. Waveguide-integrated **mid-infrared photodetection using graphene** on a scalable chalcogenide glass platform. *Nature Communications*, *13*(1), p.3915.
12. Ren, Z., Zhang, Z., Wei, J., Dong, B. and Lee, C., 2022. Wavelength-multiplexed hook nanoantennas for machine learning enabled **mid-infrared spectroscopy**. *Nature Communications*, *13*(1), p.3859.
13. Huang, K., Fang, J., Yan, M., Wu, E. and Zeng, H., 2022. Wide-field **mid-infrared single-photon upconversion imaging**. *Nature communications*, *13*(1), p.1077.
14. Hamm, M., Grott, M., Senshu, H., Knollenberg, J., de Wiljes, J., Hamilton, V.E., Scholten, F., Matz, K.D., Bates, H., Maturilli, A. and Shimaki, Y., 2022. **Mid-infrared emissivity** of partially dehydrated asteroid (162173) Ryugu shows strong signs of aqueous alteration. *Nature Communications*, *13*(1), p.364.

The structure that we demonstrate is new for QCLs and the mid-IR spectral range in particular and is a key milestone en route to more complex architectures and demonstrations of previously unseen effects, such as Nozaki-Bekki solitons, that we showed recently in the same type of device (Opačak, N., Kazakov, D., Columbo, L.L., Beiser, M., Letsou, T.P., Pilat, F., Brambilla, M., Prati, F., Piccardo, M., Capasso, F. and Schwarz, B., 2023. Nozaki-Bekki optical solitons. *arXiv preprint arXiv:2304.10796*.). Furthermore, contrary to the claim of Reviewer #2 that the fast gain dynamics is not relevant to the results presented in the current manuscript, it is precisely the fast gain dynamics that enables frequency comb generation and phase-locking in ring QCLs, the proof of which is now included in the revised version of the manuscript.

Though I could not support publication in Nature Communications, for informational purposes I would suggest that the authors address the following questions and comments to improve their manuscript:

a. The claims of nonlinear frequency conversion are not proven. The peak that is alleged to be an idler is orders of magnitude lower than the pump & signal and were measured using FTIR, which means that a large range of nonlinear artifacts could create the same effect: detector saturation, quantization artifacts, etc. Additiionally, the leftmost spectrogram in Fig. 3b seems to show that the peak is present over the whole

detuning range, which would indicate that it is not dependent on coupling into the ring. To conclusively establish that the peak alleged to be an idler is real, the authors should spectrally filter out the pump and signal, e.g. by using a grating as in Applied Physics Letters 102, 222104 (2013).

In the reference cited by the referee the authors used a grating spectrometer, not a designated grating to specifically rule out the possibility of an artifact. While we could do that, as suggested by the referee — use a grating to spatially separate the spectral lines — we resort to a method that makes direct use of active ring resonators, supporting our vision laid out in the manuscript, for the future usefulness of such devices. **We utilize a second ring resonator below its lasing threshold as a spectral notch filter** to selectively suppress the signal sideband without affecting the idler. The result is shown in the figure below and further confirms the nonlinear frequency conversion in our system.

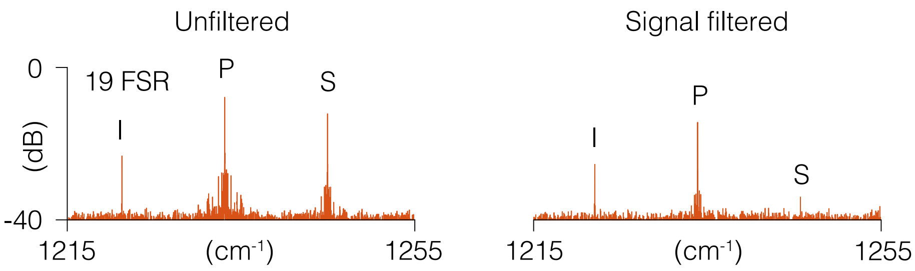

b. Are the high-frequency fluctuations evident in the detuning plots of Fig. 2a noise or actual signal? If they are noise, is there any particular reason that the SNR of these measurements is relatively low? Generally one would expect higher SNR with a powerful QCL and a HgCdTe detector.

The source of those frequency fluctuations is technical measurement noise. We have purposefully attenuated the probe beam to remain in the small signal limit and avoid gain saturation of the racetrack resonator — which becomes particularly relevant in the strongly overcoupled regimes, above the transparency point (not shown in the figure). For this reason, we used lock-in detection in this measurement. The lock-in filter cutoff was set to around 100 Hz, evidently too high to filter out the noise spotted by the referee. To better illustrate this point, below is a series of racetrack transmission measurements across the resonance for different power levels of the probe beam in two conditions: below transparency point and above transparency point. Each transmission curve is normalized by its maximum.

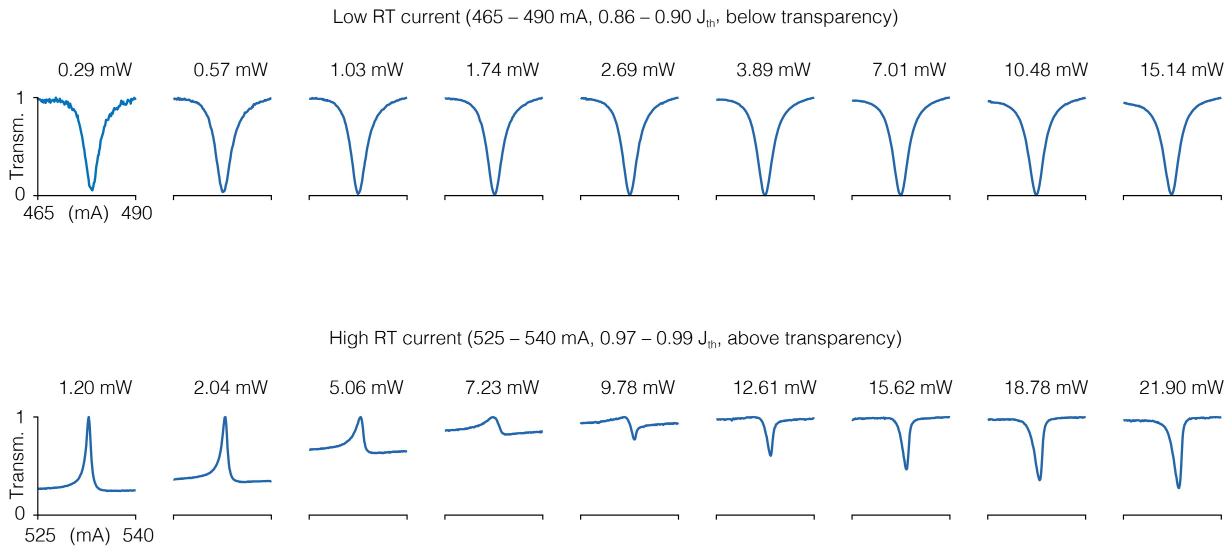

c. Is the spectrum shown in Fig. 4c truly a comb, or merely comb-like?

The spectrum presented in our manuscript is a frequency comb, as evidenced by a narrow intermode beatnote, measurement of which we now include in Fig. 4, as well as by coherent beatnote spectroscopy (SWIFTS) that we carried out on an identical state in a similar device, now included in the Supplementary Section V.

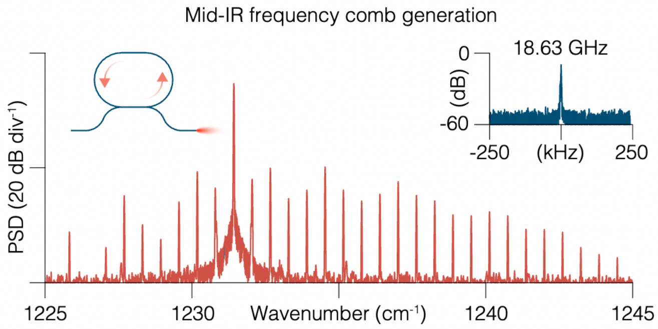

d. In my opinion, the first paragraph of the introduction is extremely misleading, as it is incredibly unlikely that this result has any relevance to data centers or to optical signal processors. There is essentially no reason to do interconnects in the mid-infrared. Basically every aspect of the chain is worse: the detectors are noisier due to lower hv/kT, the lasers are more expensive to manufacture and have lower efficiency, the fibers have higher losses and are more expensive, etc. This paragraph reads like an attempt to entice a general audience, but it does so by misleading them.

We believe that the first paragraph is not misleading, as it does not indicate an aspiration to use the demonstrated devices for optical interconnects, nor does such application follow from anywhere else in the manuscript. All possible use cases are listed in the abstract (spectroscopy, communication, microwave generation) and are described in more detail in the concluding section of the main text. It is indeed meant as a general introduction on PICs, intended to entice a general audience, by providing familiar context, which we find appropriate for the first paragraph of the manuscript.

Reviewer #3 (Remarks to the Author):

The Authors present a solid experimental study on active mid-infrared racetrack resonators based on quantum-cascade material. The layout of the proposed device is surely not new since many of these devices have been widely investigated lately in integrated optics with PIC.s. The novelty is that here the material of both racetrack and waveguide is active, allowing for a dynamical change of the coupling characteristics by acting on the pumping of the different elements.

The investigation is well conducted, and the results are surely interesting, even though I am not completely convinced they are fully matching the required general interest for Nature Comms. audience. There are some points that I would like the Authors to clarify/change.

We appreciate the positive evaluation of our work by Reviewer #3 and the singling out the novel aspects of it.

1. In terms of references, in the QCL community there have been already a few implementations of PICs. Particularly, one experiment that is very similar to the one presented is described in the paper from Kundu, I. et al. Terahertz photonic integrated circuit for frequency tuning and power modulation. Opt. Express 28, 4374–4386. (2020). I would suggest to cite also this work.

We thank Referee #3 for bringing up this work, which we indeed recognize as relevant and now include the citation of it.

This paper now appears as reference [13] in the main text of the manuscript.

2. The paper lacks a few quantitative informations: since it is integrated optics, it would be useful to know the actual value of current and applied bias (and not only current density) to evaluate the power load of the proposed device, both for Racetrack and Waveguide. Also the FSR of the racetrack and the coupler waveguide is missing from the paper.

We included the information about the length of the coupler and the FSR of the ring resonator in the Methods section, which together with the waveguide dimensions allows

conversion to current from current density. We also added operational voltage and current ranges in the Methods section.

3. Regarding the comb operation, there is little discussion about the actual coherence of the modes presented in Fig 4 c. Ideally it would be necessary to show SWIFTs or dual-comb signals to actually prove the comb nature of the laser emission. In this case, since there is an analysis of the symmetry breaking and a measurement of the CW and CCW light emission, it would be sufficient to show an optical or electrical beatnote (should be around 15 GHz so not so high in frequency...quite easy to measure.). I would say that a beat note measurement is a necessary requirement to affirm that the RT is producing a comb state.

We performed additional SWIFTS characterization of an RT QCL, following the suggestion of Reviewer #3. In Supplementary Section V we now include the experimental data obtained from a device, identical to that reported in the main text of the manuscript, showing its optical spectrum and its SWIFTS spectrum. This measurement unambiguously verifies the phase-coherence of the multimode state generated by RT QCL. We will not make any comments about the temporal waveform that this state corresponds to — complete theory and extended characterization of states generated by these devices can be found in the preprint, currently under revision (Opačak, N., Kazakov, D., Columbo, L.L., Beiser, M., Letsou, T.P., Pilat, F., Brambilla, M., Prati, F., Piccardo, M., Capasso, F. and Schwarz, B., 2023. Nozaki-Bekki optical solitons. *arXiv preprint arXiv:2304.10796*.).

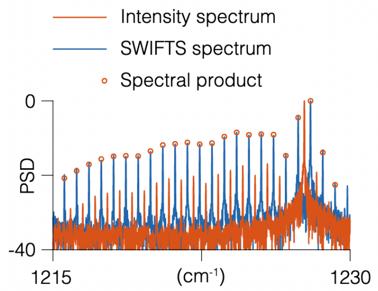

Furthermore, we update the Fig. 4 of the main text to include an RF spectrum of the beat note at the roundtrip frequency of the RT, showing sub-kHz linewidth within a 1 s sweep time of the spectrum analyzer, indicating the high coherence of the state.

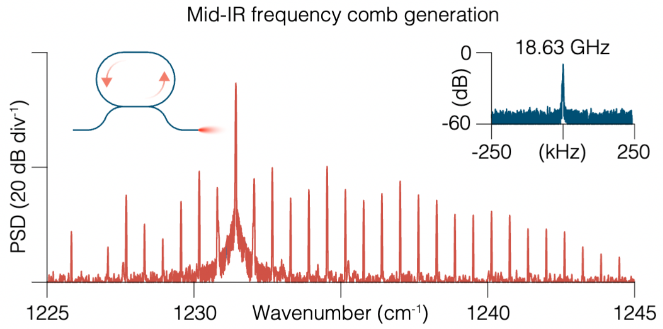

4. Still on the fig4c. is the envelope of the modes sech^2 ? otherwise is really not clear why this should be a comb

Whether the envelope of the spectrum can be fit with a sech^2 function (i.e., whether it is a soliton solution of a form of a nonlinear Schrodinger equation) is still an open question in the community. Since we are not focusing on the origins of the single-mode instability that leads to comb formation, fitting a sech^2 envelope within the context of current work would be speculative. There is a number of works attempting to shed light on the why ring QCLs generate comb states, and more are underway:

1. Opačak, N., Kazakov, D., Columbo, L.L., Beiser, M., Letsou, T.P., Pilat, F., Brambilla, M., Prati, F., Piccardo, M., Capasso, F. and Schwarz, B., 2023. Nozaki-Bekki optical solitons. *arXiv preprint arXiv:2304.10796*.
2. Meng, B., Singleton, M., Hillbrand, J., Franckié, M., Beck, M. and Faist, J., 2022. Dissipative Kerr solitons in semiconductor ring lasers. *Nature Photonics*, *16*(2), pp.142-147.
3. Kazakov, D., Opačak, N., Beiser, M., Belyanin, A., Schwarz, B., Piccardo, M. and Capasso, F., 2021. Defect-engineered ring laser harmonic frequency combs. *Optica*, *8*(10), pp.1277-1280.
4. Piccardo, M., Schwarz, B., Kazakov, D., Beiser, M., Opačak, N., Wang, Y., Jha, S., Hillbrand, J., Tamagnone, M., Chen, W.T. and Zhu, A.Y., 2020. Frequency combs induced by phase turbulence. *Nature*, *582*(7812), pp.360-364.
5. Meng, B., Singleton, M., Shahmohammadi, M., Kapsalidis, F., Wang, R., Beck, M. and Faist, J., 2020. Mid-infrared frequency comb from a ring quantum cascade laser. *Optica*, *7*(2), pp.162-167.

In conclusion, the Authors should produce more convincing data to substantiate the claim of comb operation.

The SWIFTS measurement and the beat note spectrum added in the revised version of the manuscript fully address this point of Reviewer #3.

REVIEWER COMMENTS

Reviewer #1 (Remarks to the Author):

The authors have addressed all of my concerns.

Reviewer #2 (Remarks to the Author):

The authors have addressed my technical comments, although I still maintain my reservations about the broader impact. It seems to me that the main impact lies in the other work that they mentioned (Opačak, N., Kazakov, D., Columbo, L.L., Beiser, M., Letsou, T.P., Pilat, F., Brambilla, M., Prati, F., Piccardo, M., Capasso, F. and Schwarz, B., 2023. Nozaki-Bekki optical solitons. arXiv preprint arXiv:2304.10796), not this work.

The section on "Ring frequency combs with active couplers" that they point to as requiring the fast gain especially seems to have some overlap with that work, as the version of Fig. 4c from the prior revision seems very similar to Fig. 2d in arXiv:2304.10796. Assuming that work is also under review, the same impact cannot be claimed here and there at the same time. At the very least it must be cited and more information should be provided to delineate the two.

Reviewer #3 (Remarks to the Author):

The Authors answered satisfactorily to my comments and added data to the manuscript that now can be accepted for publication.

- Reviewer comments are in red, shown without editing, as they appeared in the original communication from the editor
- Our responses are in blue
- Summaries of the implemented changes are in green

REVIEWER COMMENTS

Reviewer #1 (Remarks to the Author):

The authors have addressed all of my concerns.

We thank the reviewer for the careful evaluation of our manuscript.

Reviewer #2 (Remarks to the Author):

The authors have addressed my technical comments, although I still maintain my reservations about the broader impact. It seems to me that the main impact lies in the other work that they mentioned (Opačak, N., Kazakov, D., Columbo, L.L., Beiser, M., Letsou, T.P., Pilat, F., Brambilla, M., Prati, F., Piccardo, M., Capasso, F. and Schwarz, B., 2023. Nozaki-Bekki optical solitons. arXiv preprint arXiv:2304.10796), not this work.

The section on "Ring frequency combs with active couplers" that they point to as requiring the fast gain especially seems to have some overlap with that work, as the version of Fig. 4c from the prior revision seems very similar to Fig. 2d in arXiv:2304.10796. Assuming that work is also under review, the same impact cannot be claimed here and there at the same time. At the very least it must be cited and more information should be provided to delineate the two.

We thank the reviewer for the further technical evaluation of the revised version. Regarding the relation of the present work to the submission by Opačak et al., we would like to emphasize that our manuscript **is not supplementary** to the results shown in the arXiv submission on Nozaki-Bekki optical solitons. The study by Opačak et al., reports an extended experimental characterization of the frequency comb states, similar to the one reported in Fig. 4 of our manuscript, as well as the temporal waveforms associated with them, and develops a theoretical framework that allows to explain their formation, which is not the focus of our manuscript. In contrast, our paper, more broadly, provides a platform for nonlinear integrated photonics in the mid-infrared and introduces the associated experimental techniques, such as injection of external coherent control signals and the ability to use the reported resonators below their lasing threshold as reconfigurable components for spectral filtering, which are the main focuses of the present submission.

We agree that we should clarify the relationship between the two works and cite the arXiv submission by Opačak et al. With this in mind we added the following sentence in the revised version of the manuscript (line 227 in the main text):

An in-depth theoretical and experimental investigation of frequency comb states and the underlying nonlinear dynamics of their formation is discussed in Ref. [53], a study made possible by the active waveguide technology presented in this paper.

Reviewer #3 (Remarks to the Author):

The Authors answered satisfactorily to my comments and added data to the manuscript that now can be accepted for publication.

We thank the reviewer for the careful evaluation of our manuscript and for the acceptance recommendation.

REVIEWER COMMENTS

Reviewer #2 (Remarks to the Author):

The authors did not address the similarity of the initially-submitted Fig. 4c and Fig. 2d of arXiv:2304.10796, another manuscript that appears to be under review. Given that these two figures are extremely similar over several orders of magnitude, including imperfections, it must be clarified if there is any relationship between the two.

In my opinion, the addition of one sentence does not solve the underlying issue, which is that the most impactful part of the manuscript overlaps with arXiv:2304.10796. Taking away the comb section, one is left with a manuscript on the platform itself, which has been performed previously in other active systems (just not in the mid-infrared with quantum cascade lasers).

- Reviewer comments are in red, shown without editing, as they appeared in the original communication from the editor.
- Our responses are in blue.
- Summaries of the implemented changes are in green

REVIEWER COMMENTS

Reviewer #2 (Remarks to the Author):

The authors did not address the similarity of the initially submitted Fig. 4c and Fig. 2d of arXiv:2304.10796, another manuscript that appears to be under review. Given that these two figures are extremely similar over several orders of magnitude, including imperfections, it must be clarified if there is any relationship between the two.

We thank the Reviewer for further assessment of the manuscript and for singling out the unclear aspects. The spectrum in Fig. 4c of the initial version of the NatComm submission and the Fig. 2d of arXiv:2304.10796 show the optical spectrum obtained from the same device under identical driving conditions, however, taken at different times. To facilitate their comparison we show them below, overlapped:

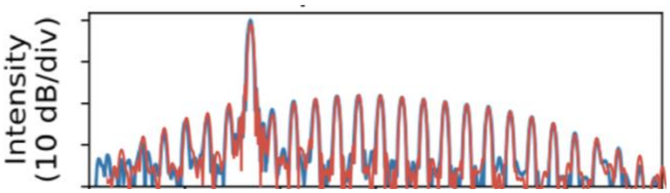

It is apparent that the two spectra do not represent the same dataset, as seen from the non-overlapping noise features between the comb lines. Over the course of the research reported in our NatComm submission we have characterized tens of devices, some of which were and are being used in subsequent research, including, among others, the investigation of Nozaki-Bekki optical solitons, the results of which are reported in arXiv:2304.10796. To illustrate the universality of the phenomenon of comb formation in RT QCLs and to show that the comb states are robustly generated in devices with the new architecture we added a Supplementary Section VI, showing optical spectra of eight more representative RT QCLs:

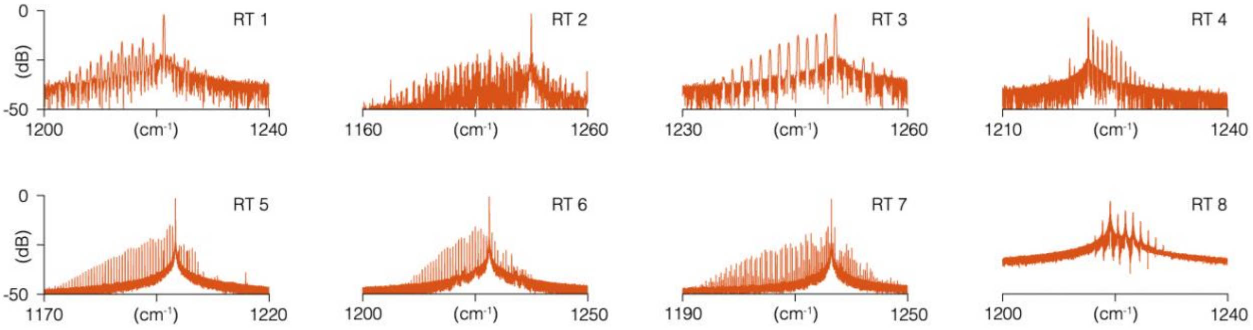

In my opinion, the addition of one sentence does not solve the underlying issue, which is that the most impactful part of the manuscript overlaps with arXiv:2304.10796. Taking away the comb section, one is left with a manuscript on the platform itself, which has been performed previously in other active systems (just not in the mid-infrared with quantum cascade lasers).

We disagree that the most impactful part of the manuscript is the comb generation in a ring QCL. The onset of research on frequency comb generation in QCLs can be traced back to the original paper by the Faist group at ETH Zurich (Hugi, A., Villares, G., Blaser, S., Liu, H.C. and Faist, J., 2012. Mid-infrared frequency comb based on a quantum cascade laser. *Nature*, *492* (7428), pp. 229-233.). The following ten years have seen many impactful works on QCL frequency combs, **including more recent investigations of frequency comb generation in monolithic ring cavity QCLs** (Refs. 46, 47, 48, 49, 51, 61). Going along with the Reviewer's logic, frequency comb generation in ring QCLs is not a novelty either, and the result of Fig. 4 of our current manuscript could no be deemed as impactful, since it has already been demonstrated previously — an argument coherent with the one that active resonators have been shown in other platforms.

In our opinion, however, **it is not an angle at which the results of our manuscript are to be viewed**. We believe that bringing the concepts that worked well in one wavelength range, into another part of the spectrum, where they have not been demonstrated before, first, is not for granted, and second, is on its own an empowering advancement. Integration of ring QCLs with active directional couplers **enables a platform for nonlinear integrated photonics in the mid-infrared wavelength range**. Shown functions — filtering, when driven below threshold, comb generation and frequency conversion above threshold — can be put all together to create new devices in the mid-infrared, such as chip-scale short pulse generators, on-chip dual-comb spectroscopy systems, integrated 2D nonlinear IR spectrometers, just to name a few. The study of arXiv:2304.10796 on Nozaki-Bekki solitons in ring QCL resonators is an excellent manifestation of the empowering potential of the platform that we show in the current manuscript. These two works are, however, to be evaluated on their own merits. To summarize, the "Active mid-infrared ring resonators" manuscript achieves the following:

1. The design, fabrication, and exhaustive systematic evaluation of racetrack QCLs with active directional couplers, with the separation gaps between the coupled waveguides as narrow as 0.8 µm, achieved with standard optical lithography.
2. The experimental demonstration of coupling of external radiation to racetrack QCLs via the directional coupler ports, and use of ring QCLs as active filters, when driven below threshold. Such tunable photonic integrated filters have not been shown in this wavelength range.
3. The experimental demonstration of unidirectional lasing in racetrack QCLs, despite the possible higher backscattering due to reflections from the waveguide coupler facets. Unidirectional operation is essential for generating soliton states, either self-starting (subject of arXiv:2304.10796), or coherently driven (theoretically proposed in Ref. 61).

4. The experimental demonstration of racetrack QCLs achieving output optical power level on par with Fabry-Perot QCLs — a milestone, that if not reached, would fundamentally prevent ring QCLs from entering applications. We show that ring QCLs are equal competitors in this regard with Fabry-Perot QCLs.
5. The experimental demonstration that light injected from an external mid-IR QCL into the racetrack QCL via the coupler port interacts with the racetrack intracavity field coherently, resulting in the generation of FWM sidebands. This experiment is essential to further investigations on coherent driving of soliton states in ring QCLs and the experimental unification of active laser resonators with passive Kerr nonlinear microresonators (the vision proposed in ref. 61).
6. Demonstrating self-starting free-running frequency comb generation in racetrack QCLs with directional couplers.

Each of these points alone would be significant enough to be reported as a separate manuscript. We forego such a strategy and instead unify these achievements under an umbrella of a new platform for active nonlinear photonics in the mid-infrared range. To reflect upon the discussion above we add the following figure and the accompanying discussion to the Supplementary Section VII portraying our vision for the future mid-IR integrated photonic devices that naturally emerge from the results reported in our current manuscript:

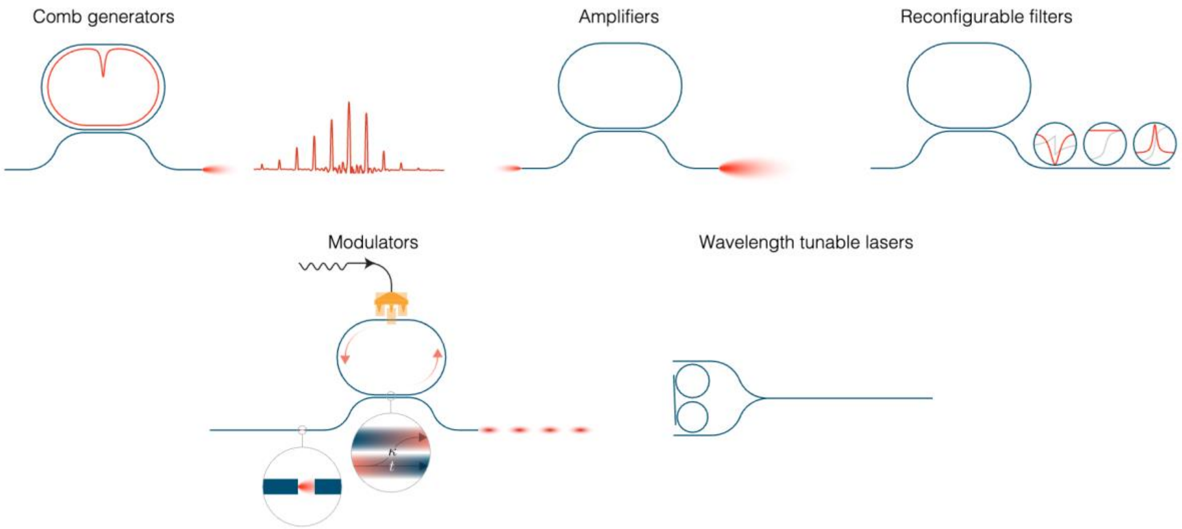

REVIEWERS' COMMENTS

Reviewer #2 (Remarks to the Author):

Thank you for clarifying the relationship between the two results further. I will defer to the editor to decide whether data taken from the same device under the same conditions is permitted within the journal's editorial policies for manuscripts under review simultaneously.
